# Targeted O‑GlcNAcylation of CK2α Triggers Its Ubiquitin-Proteasome Degradation and Alters Downstream Phosphorylation

**DOI:** 10.1021/acschembio.5c00223

**Published:** 2025-06-16

**Authors:** Tongyang Xu, Bowen Ma, Yuanpei Li, Zhihao Guo, Miaomiao Zhang, Billy Wai-Lung Ng

**Affiliations:** † Guangdong-Hong Kong-Macao Joint Laboratory for New Drug Screening, School of Pharmacy, The Chinese University of Hong Kong, Sha Tin, Hong Kong; ‡ Li Ka Shing Institute of Health Sciences, Faculty of Medicine, The Chinese University of Hong Kong, Sha Tin, Hong Kong; § Gerald Choa Neuroscience Institute, The Chinese University of Hong Kong, Sha Tin, Hong Kong; ∥ Peter Hung Pain Research Institute, Faculty of Medicine, The Chinese University of Hong Kong, Sha Tin, Hong Kong

## Abstract

O-Linked β-*N*-acetylglucosamine-modification (O-GlcNAcylation) is an important post-translational modification (PTM), yet dissecting its protein-specific functions has remained challenging. Here, we applied our previously reported chemical biology tool, the O-GlcNAcylation Targeting Chimera (OGTAC), to specifically induce O-GlcNAcylation of the casein kinase II subunit α (CK2α) at Ser347 in living cells. We found that this targeted O-GlcNAcylation destabilized CK2α through ubiquitin-proteasome degradation and enhanced its interaction with cereblon (CRBN). Overexpression and knockdown experiments also indicated CK2α as a substrate of the Cullin-RING E3 ubiquitin ligase 4-CRBN (CRL4^CRBN^) E3 ligase complex. Furthermore, the OGTAC-induced O-GlcNAcylation of CK2α reprogrammed phosphorylation of Akt and PFKP. These findings reveal that a single O-GlcNAc modification can serve as a molecular switch, controlling the protein stability and downstream phosphorylation of CK2α. More broadly, our results highlight the profound utility of the OGTAC-mediated O-GlcNAcylation to interrogate its cellular functions with specificity, overcoming limitations inherent to prior global perturbation methods.

## Graphical abstract

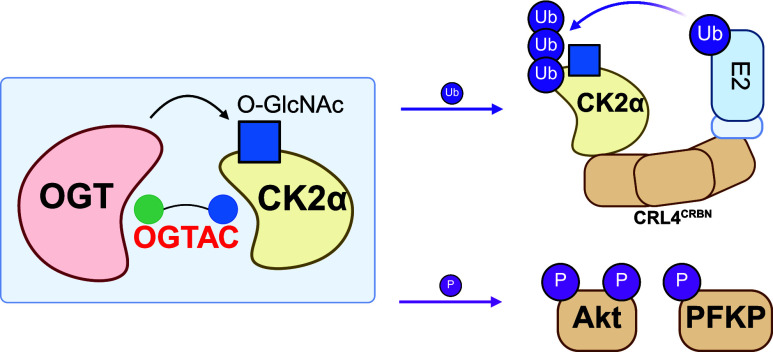

## Introduction

O-GlcNAcylation, the addition of O-linked β-*N*-acetylglucosamine (O-GlcNAc) to serine/threonine residues, is a dynamic post-translational modification (PTM) that regulates thousands of intracellular proteins and impacts diverse cellular processes, including signaling, metabolism, and transcription.^1^ Dysregulation of O-GlcNAcylation is implicated in numerous diseases such as cancer, diabetes, and neurodegeneration.^2–4^

However, a fundamental obstacle in research of O-GlcNAcylation has been the lack of tools to precisely control this PTM of individual proteins. Conventional approaches to modulate O-GlcNAcylation level involve global inhibition of the enzymes responsible, which are O-GlcNAc transferase (OGT) and O-GlcNAcase (OGA).^1,5^ However, these approaches broadly perturb cellular O-GlcNAcylation patterns, obscuring direct functional consequences for individual substrates. This limitation has hindered a clear understanding of how specific O-GlcNAc modifications alter protein stability, interactions, and signaling functions.

To overcome these challenges, we recently developed O-GlcNAcylation Targeting Chimeras (OGTACs).^6^ OGTACs are bifunctional small molecules that specifically recruit OGT to target proteins in living cells, enabling protein-specific O-GlcNAcylation. Complementing other chemical biology approaches,^7–9^ OGTACs provide unparalleled precision, enabling direct interrogation of cause-and-effect relationships between O-GlcNAcylation and protein function, without the ambiguity inherent to global perturbation tools.

Here, by targeting casein kinase II subunit α (CK2α), we illustrate the utility of OGTAC. CK2α is an essential serine/threonine kinase critical for cellular signaling and implicated in multiple pathologies including cancers and Alzheimer’s disease.^10,11^ It phosphorylates hundreds of substrates,^10,12^ including activation of Akt, which drives PI3K/Akt pathway dysregulation and tumorigenesis.^13,14^ Notably, CK2α is overexpressed in multiple cancer cell lines, such as the cervical carcinoma HeLa cells and the colon cancer HCT116 cells.^15,16^ Besides, CK2α is modified by multiple PTMs, including phosphorylation at Thr344, Thr360, Ser362, and Ser370, as well as O-GlcNAcylation at Ser347 (Figure 1A,B).^8,17,18^

**Figure 1:**
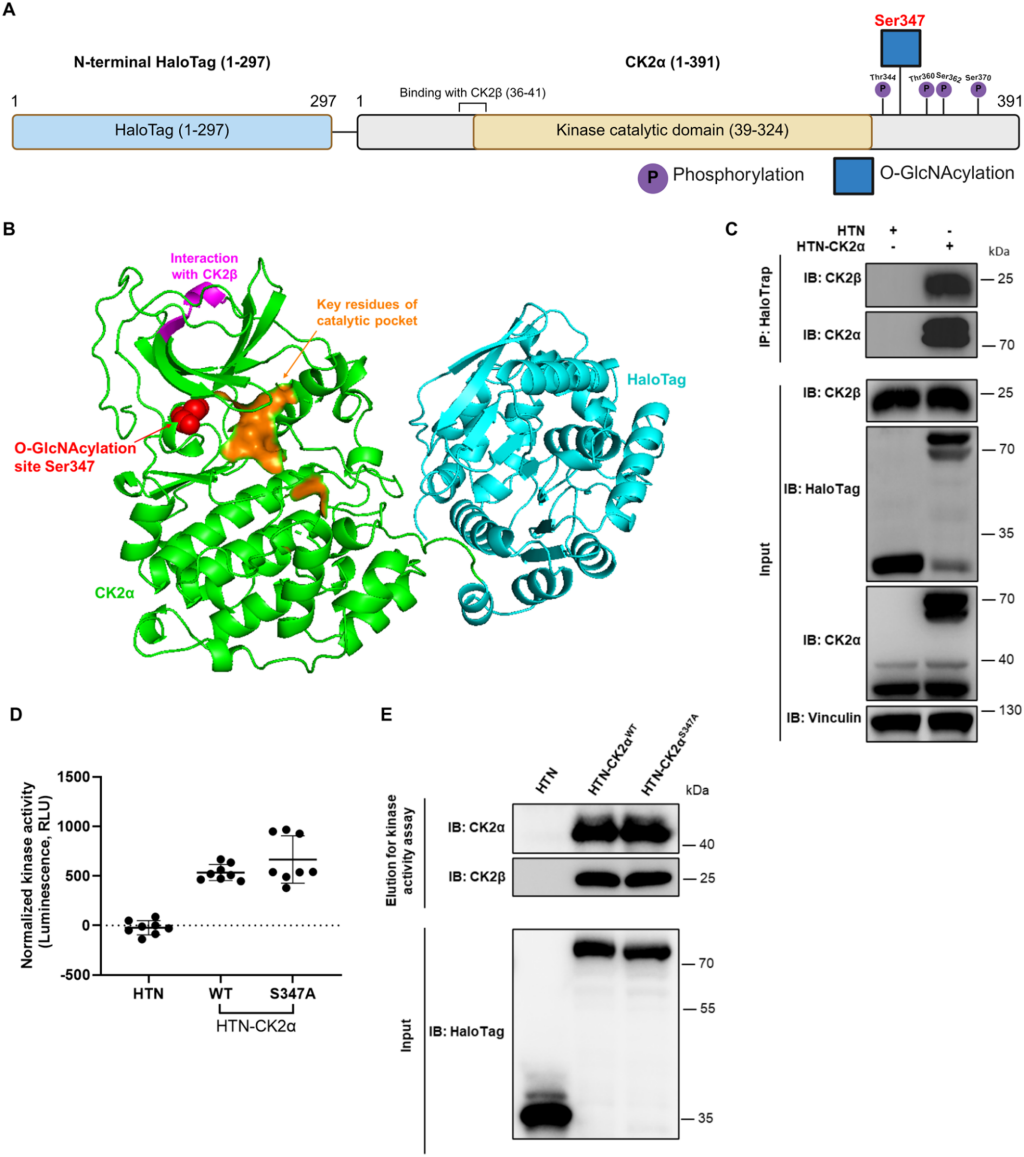
CK2α structure and kinase activity validation. (A) Schematic diagram of the domains and PTM sites of HTN-CK2α. (B) Structure modeling of HTN-CK2α to illustrate the spatial arrangement of the HaloTag, CK2α, the Ser347 O-GlcNAcylation site, key residues of the catalytic pocket, and the region interacting with CK2β. (C) HTN-CK2α interaction with endogenous CK2β. HeLa cells were transfected with either HTN-CK2α or HTN. HaloTrap was used for IP, and eluted proteins were analyzed by Western blot. (D) Both HTN-CK2α and HTN-CK2α^S347A^ retained kinase catalytic activity phosphorylating a known CK2 substrate (RRRDDD**S**DDD), detected by the ADP-Glo assay. Error bars represent the mean (SD) from *n* = 8 technically independent experiments. (E) Characterization of the purified HTN and HTN-CK2α (S347A or WT). They were individually overexpressed in HeLa and purified by the Halo-Link resin. The cleavage elution was used for the ADP-Glo assay in (D).

For CK2α O-GlcNAcylation, prior studies using semisynthesis and microinjection suggested that S-GlcNAc modification at Ser347 modulates substrate selectivity and antagonizes Thr344 phosphorylation, potentially promoting proteasomal degradation.^19,20^ Proteomic analyses further revealed CK2α O-GlcNAcylation impacts global phosphoproteome.^8^ However, the mechanisms by which CK2α O-GlcNAcylation crosstalks with ubiquitination and influences substrate specificity remain unclear. Additionally, the precise mechanism underlying CK2α degradation has yet to be elucidated. Clarifying these points will significantly advance our understanding of the regulatory roles of O-GlcNAcylation.

Leveraging our OGTAC platform, we specifically induced CK2α O-GlcNAcylation at Ser347 in living cells, providing definitive insight into the biological consequences of this modification. We demonstrate that targeted O-GlcNAcylation triggers CK2α ubiquitination and proteasomal degradation as a substrate of the Cullin-RING E3 ubiquitin ligase 4-cereblon (CRL4^CRBN^) E3 complex and alters downstream phosphorylation patterns of Akt and PFKP. These findings elucidate the crosstalk between CK2α O-GlcNAcylation and its ubiquitination, along with the altered downstream phosphorylation, resolving previous controversies and uncovering new mechanistic insights.

## Results and Discussion

### OGTAC-1 Specifically Induces CK2α O-GlcNAcylation in Living Cells

First, we validated the kinase activity of the N-terminal HaloTag-CK2α (HTN-CK2α) that we used in this study. Structure modeling of HTN-CK2α indicated that the N-terminal HaloTag (HTN) did not spatially obstruct the CK2α catalytic domain, the region interacting CK2β, or the Ser347 O-GlcNAcylation site (Figure 1B). This provided initial evidence that the tag did not interfere with the protein function. Furthermore, CK2α typically functions by forming a complex with CK2β.^21^ We found that the overexpressed HTN-CK2α can normally interact with CK2β, while the overexpressed HTN alone cannot (Figure 1C). Additionally, both HTN-CK2α and HTN-CK2α^S347A^ (Ser347 O-GlcNAcylation-site mutant) retained kinase catalytic activity, effectively phosphorylating a known CK2 substrate (RRRDDD**S**DDD) in the *in vitro* ADP-Glo assay (Figure 1D,E).^22^ These results further supported the use of HTN-CK2α for CK2α functional studies.

To verify the OGTAC-1 effects of O-GlcNAcylation induction on CK2α in living HeLa cells, we first optimized co-overexpression of FKBP12^F36V^-2 × HA-OGT (fOGT) and HTN-CK2α (Supporting Figure 1A), and plasmid ratio at HTN-CK2α/fOGT = 1:0.2. At this ratio, both proteins were successfully expressed and clearly detected by Western blot, where the O-GlcNAcylation level of HTN-CK2α and the whole proteome stayed unchanged (Supporting Figure 1A,B).

With the expression of fOGT and HTN-CK2α, we applied OGTAC-1 at various concentrations ranging from 0.1 to 10,000 nM to induce the O-GlcNAcylation of HTN-CK2α. OGTAC-1 is a bifunctional molecule that targets fOGT and HTN-CK2α, increasing their proximity and inducing CK2α O-GlcNAcylation. Through immunoprecipitation (IP) assays, and using RL2 antibody to detect O-GlcNAcylation, we found that O-GlcNAcylation level of HTN-CK2α increased by about 2-fold at 8 h with 100 nM OGTAC-1 (Figure 2B,C). Hook effect of OGTAC-1 was observed. Treating OGTAC-1 did not affect CK2α-CK2β interaction (Figure 2B).

**Figure 2:**
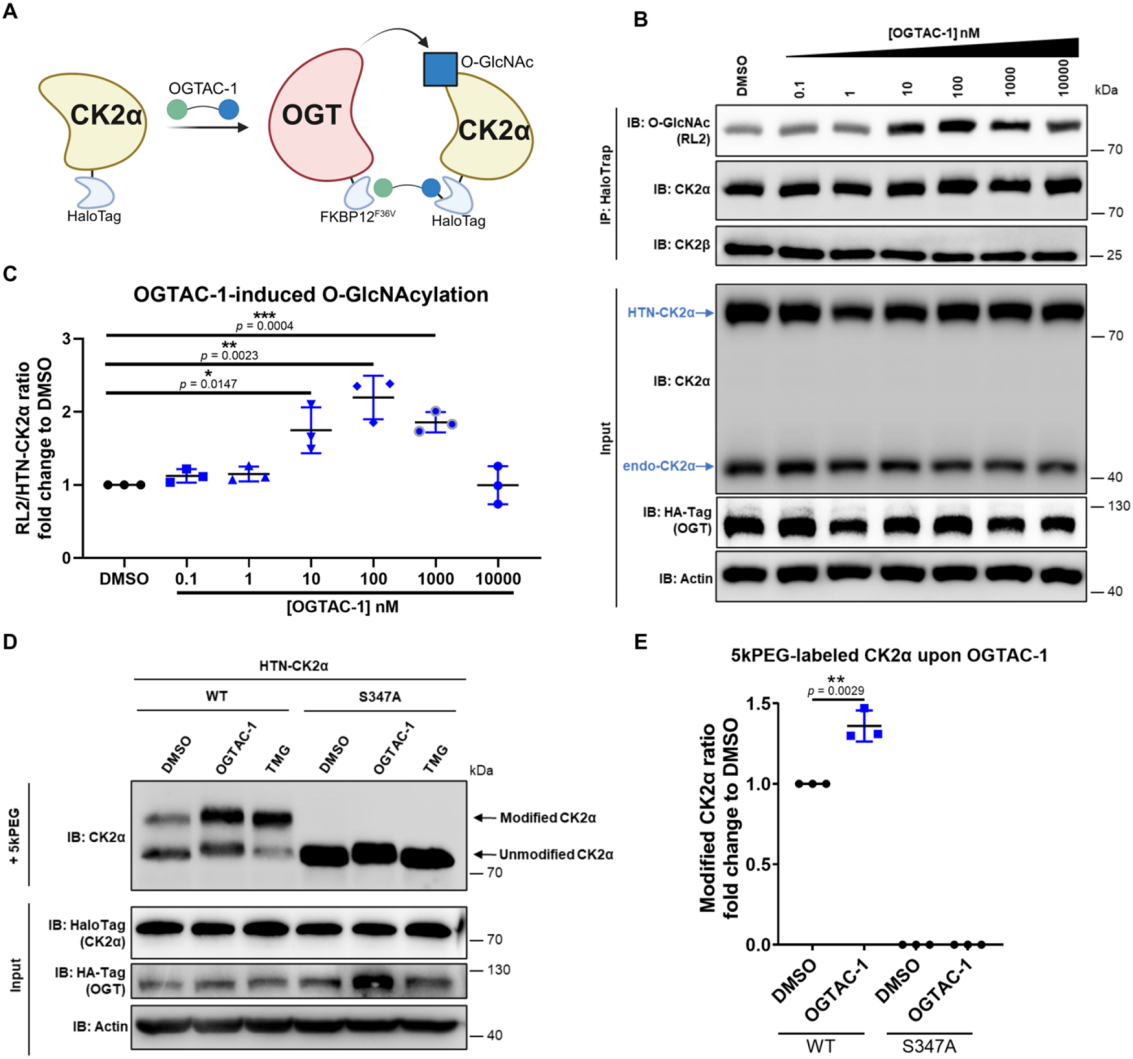
OGTAC-1 specifically induced CK2α Ser347 O-GlcNAcylation. (A) Schematic diagram of using OGTAC-1 to induce CK2α O-GlcNAcylation. (B) OGTAC-1 induced targeted O-GlcNAcylation of CK2α at various concentrations (0.1 to 10,000 nM, every 10-fold) for 8 h of treatment, with a maximum 2-fold induction and hook effect. The O-GlcNAcylation of HTN-CK2α was assessed by immunoblotting after IP using HaloTrap. (C) Quantification of relative HTN-CK2α O-GlcNAcylation (RL2 signal normalized to CK2α IP) fold changes over DMSO from panel (B). Error bars represent the mean (SD) from *n* = 3 biologically independent experiments. (D) OGTAC-1 specifically induced HTN-CK2α^WT^ Ser347 O-GlcNAcylation by about 1.5-fold at 100 nM for 8 h, while HTN-CK2α^S347A^ did not show O-GlcNAcylation after treatment with OGTAC-1. (E) Quantification of relative upshifted CK2α (upshifted CK2α normalized to total CK2α) over DMSO from panel (D). Error bars represent the mean (SD) from *n* = 3 biologically independent experiments. Statistical significance was assessed using an unpaired Student’s *t*-test. *p < 0.05; **p < 0.01; ***p < 0.001. All schematics in this paper were created *via* BioRender (https://biorender.com).

Then, we further validated that the OGTAC-1 induced targeted O-GlcNAcylation through recruiting the catalytically active fOGT instead of stimulating cell stress. We applied OGTAC-1 to cells that only overexpressed HTN-CK2α, or co-overexpressed HTN-CK2α and fOGT^K852A^ (Supporting Figure 2A,B). fOGT^K852A^ is the inactive form of OGT, which lacks the catalytic activity to induce O-GlcNAcylation.^23^ O-GlcNAcylation of HTN-CK2α did not significantly changed upon OGTAC-1 treatment, with or without fOGT^K852A^. This indicated that OGTAC-1 did not raise the O-GlcNAcylation level of HTN-CK2α by stimulating endogenous OGT *via* cell stress but through direct OGT catalytic activity.

Previously, it was reported that Ser347 is the only O-GlcNAcylation site of CK2α. We tested whether OGTAC-1 induced any new O-GlcNAcylation sites on CK2α. We conducted GalT enzymatic labeling followed by PEGylation (using PEG5k), which can result in an upshift of molecular weight for the O-GlcNAcylated proteins (Figure 2D,E). The O-GlcNAcase (OGA) inhibitor thiamet G (TMG), a global O-GlcNAcylation modulator, was used as a positive control to globally increase O-GlcNAcylation level.^24^ The results showed increased O-GlcNAcylated HTN-CK2α^WT^ by about 1.5-fold upon OGTAC-1 treatment at 8 h comparing to DMSO, while HTN-CK2α^S347A^ totally lost O-GlcNAcylation. These results indicate that OGTAC-1 induced CK2α O-GlcNAcylation only at Ser347. Our previous studies also showed that OGTAC-1 only induced O-GlcNAcylation of HTN-CK2α, but not endogenous CK2α.^6^ These suggest both site- and target-specificity of OGTAC-1 for CK2α.

### Targeted O-GlcNAcylation Triggers CK2α Ubiquitination and Proteasomal Degradation

Using OGTAC-1 to specifically induce CK2α O-GlcNAcylation, we investigated changes in CK2α stability. First, we assessed CK2α degradation over time using a cycloheximide (CHX) chase assay (Figure 3A,B). OGTAC-1 treated HTN-CK2α^WT^ represented a higher level of O-GlcNAcylation than DMSO, while the HTN-CK2α^S347A^ lacked O-GlcNAcylation, with or without OGTAC-1 (Figure 2B–E). As the O-GlcNAcylation level of CK2α increased, its degradation significantly accelerated (Figure 3A,B). Also, in a 12 h CHX chase assay following an 8 h pretreatment of OGTAC-1, only wildtype HTN-CK2α showed a significantly faster degradation in both HeLa and HCT116 cells, compared to the DMSO controls (Figure 3C,D; and Supporting Figure 3A,B). This indicates that OGTAC-1 accelerates CK2α degradation across different cancer cell lines. Notably, inducing proximity between fOGT and HTN-CK2α^S347A^ by OGTAC-1 did not change its stability, which highlights that the effect is dependent on CK2α S347 O-GlcNAcylation.

**Figure 3:**
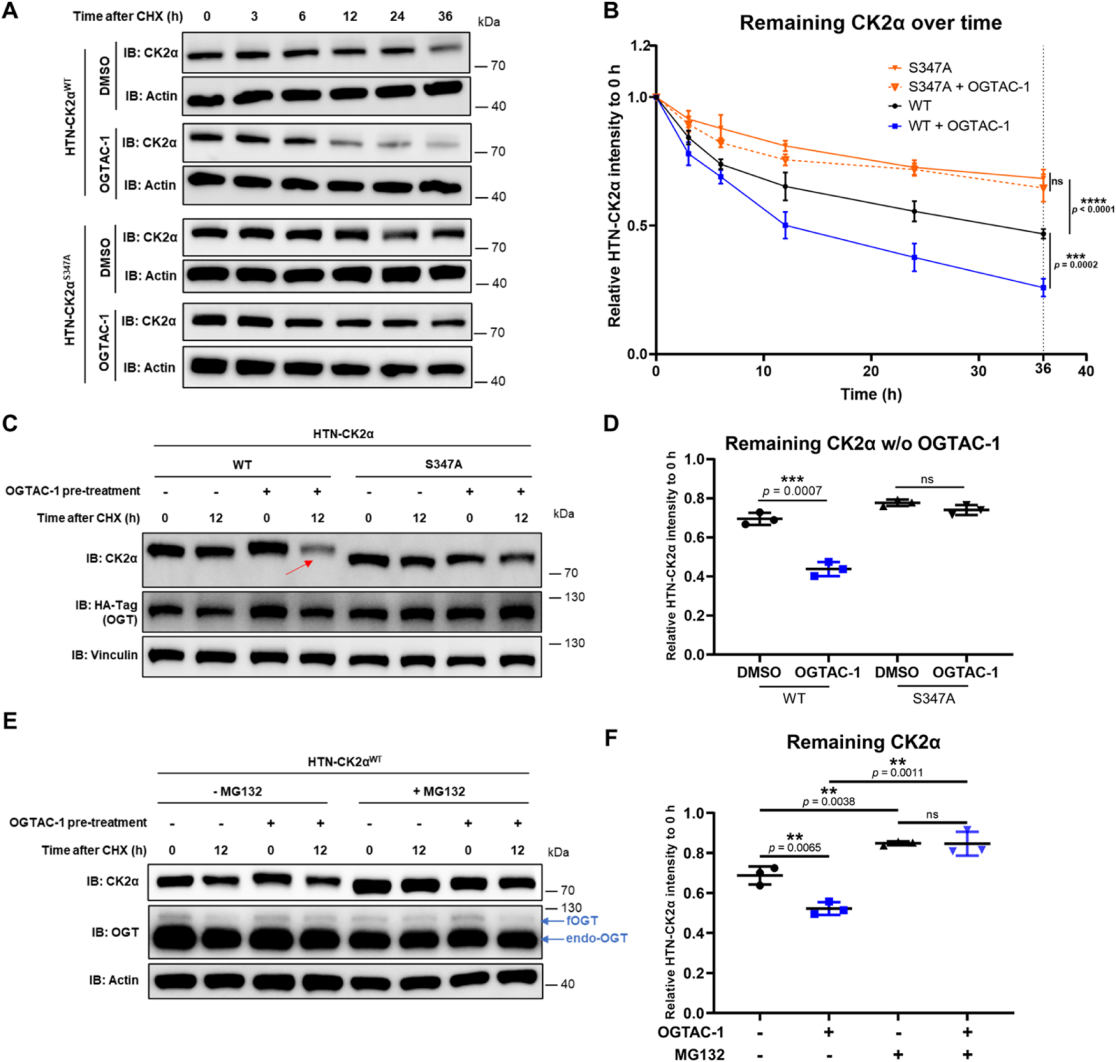
Targeted O-GlcNAcylation accelerates CK2α proteasomal degradation. (A) 36 h CHX chase assay of HTN-CK2α (WT or S347A) to determine the degradation rate of HTN-CK2α following pretreatment of OGTAC-1 (1 μM) or DMSO. (B) Quantification of the remaining relative CK2α level (CK2α normalized to Actin) at each time point (0/3/6/12/24/36 h) of CHX treatment in (A), normalized to the 0 h level. Error bars represent the mean (SD) from *n* = 3 biologically independent experiments. (C) After adding CHX for 12 h, only HTN-CK2α^WT^ with OGTAC-1 (1 μM) pretreatment showed significantly more degradation. (D) Quantification of remaining relative CK2α after 12 h CHX treatment in (C), as described above. (E) The proteasome inhibitor MG132 increased the CK2α level before and after the 12 h CHX treatment, with or without OGTAC-1 pretreatment. (F) Quantification of the remaining relative CK2α after 12 h CHX treatment in (E), as described above. Error bars represent the mean (SD) from *n* = 3 biologically independent experiments. Statistical significance in panel (B) was assessed using a two-way ANOVA. Statistical significance in (D) and (F) was assessed using an unpaired Student’s *t*-test. *p < 0.05; **p < 0.01; ***p < 0.001; ns, not significant.

Then, we found that the proteasome inhibitor MG132 can rescue CK2α degradation regardless of the presence of OGTAC-1 (Figure 3E,F). Yet treating the lysosomal degradation inhibitor chloroquine (CQ) did not show such effects (Supporting Figure 3C,D). These revealed that both CK2α degradation, and the degrading acceleration induced by OGTAC-1, occurred *via* the proteasomal degradation pathway. This is consistent with previous studies showing that MG132 can stabilize the S-GlcNAc-modified CK2α.^19^

As proteasomal degradation is closely related to ubiquitination, we wonder whether targeted O-GlcNAcylation of CK2α can affect its ubiquitination level.^25,26^ We examined changes in CK2α ubiquitination level following alterations in O-GlcNAcylation, with 2 h MG132 pretreatment to enrich ubiquitination on CK2α. Significantly, around 2-fold higher levels of O-GlcNAcylation on CK2α induced by 8 h of OGTAC-1 treatment resulted in a nearly 1.5-fold increase in CK2α ubiquitination (Figures 2B,C and 4A). A significant decrease was observed for ubiquitination of the O-GlcNAcylation-deficient CK2α, with or without OGTAC-1 treatment. This indicates that targeted O-GlcNAcylation triggers CK2α degradation through ubiquitin-proteasome system (UPS).

**Figure 4:**
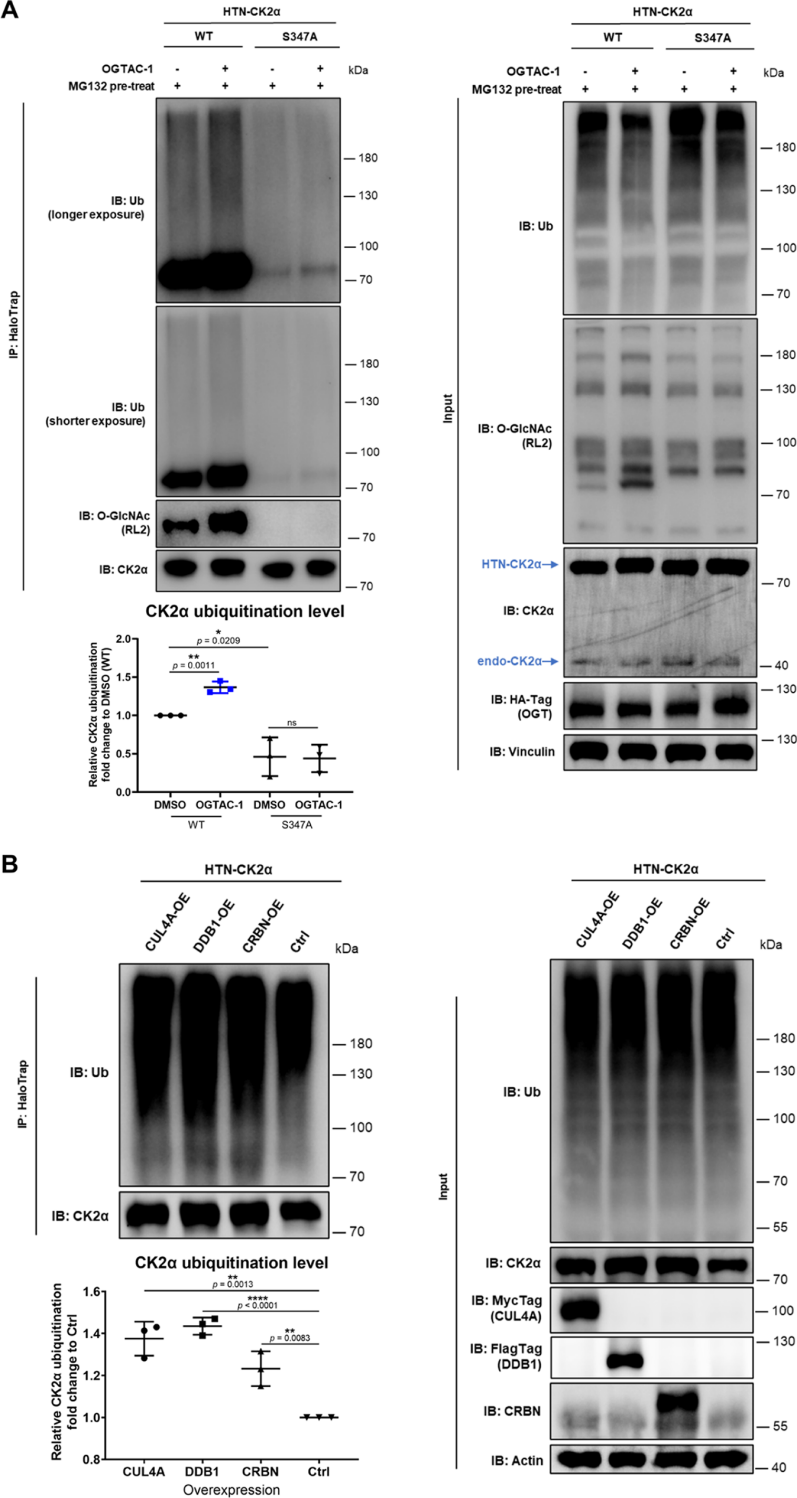
Targeted O-GlcNAcylation and overexpression of CRL4^CRBN^ E3 components increase CK2α ubiquitination. (A) Targeted O-GlcNAcylation of CK2α after 8 h of OGTAC-1 treatment led to its enhanced ubiquitination and quantification. Relative CK2α ubiquitination level (ubiquitination signal normalized to CK2α IP) was normalized to that of the DMSO-treated HTN-CK2α^WT^ as fold changes. (B) Overexpressing any of CUL4A/DDB1/CRBN increased CK2α ubiquitination, and quantification. Relative CK2α ubiquitination level (ubiquitination signal normalized to CK2α IP) was normalized to that of the empty vector control group as the fold changes. Error bars represent the mean (SD) from *n* = 3 biologically independent experiments. Statistical significance was assessed using an unpaired Student’s *t*-test. *p < 0.05; **p < 0.01; ****p < 0.0001; ns, not significant.

### CK2α Is a Substrate of the CRL4^CRBN^ E3 Ubiquitin Ligase Complex

Next, we investigated which specific E3 ligases could be involved in the proteasomal degradation of CK2α. Previous studies have reported that CK2α interacted with CUL4A and DDB1 *in planta*, and that DDB1 destabilized CK2α *in vitro*.^27^ A prior study degraded CK2α by recruiting CRBN.^28^ CUL4A-DDB1-CRBN is a known E3 complex (CRL4^CRBN^), where CRBN plays as the substrate receptor protein.^29^ We hypothesized and tested *in cellulo* whether CK2α is a substrate of this E3 complex.

First, by overexpressing any of the three CRL4^CRBN^ components, we detected a significant increase in CK2α ubiquitination, suggesting that they are directly involved in CK2α ubiquitination (Figure 4C,D).

Then, we performed coimmunoprecipitation (co-IP) experiments to verify the protein–protein interactions. Co-overexpression of HTN-CK2α with Flag-DDB1, Myc-CUL4A, or Flag-CRBN in living cells allowed us to detect co-IP of the CRL4^CRBN^ components respectively with HTN-CK2α, but not with HTN (Figure 5A–C). These results confirmed the direct interactions between CK2α and the CRL4^CRBN^ components.

**Figure 5:**
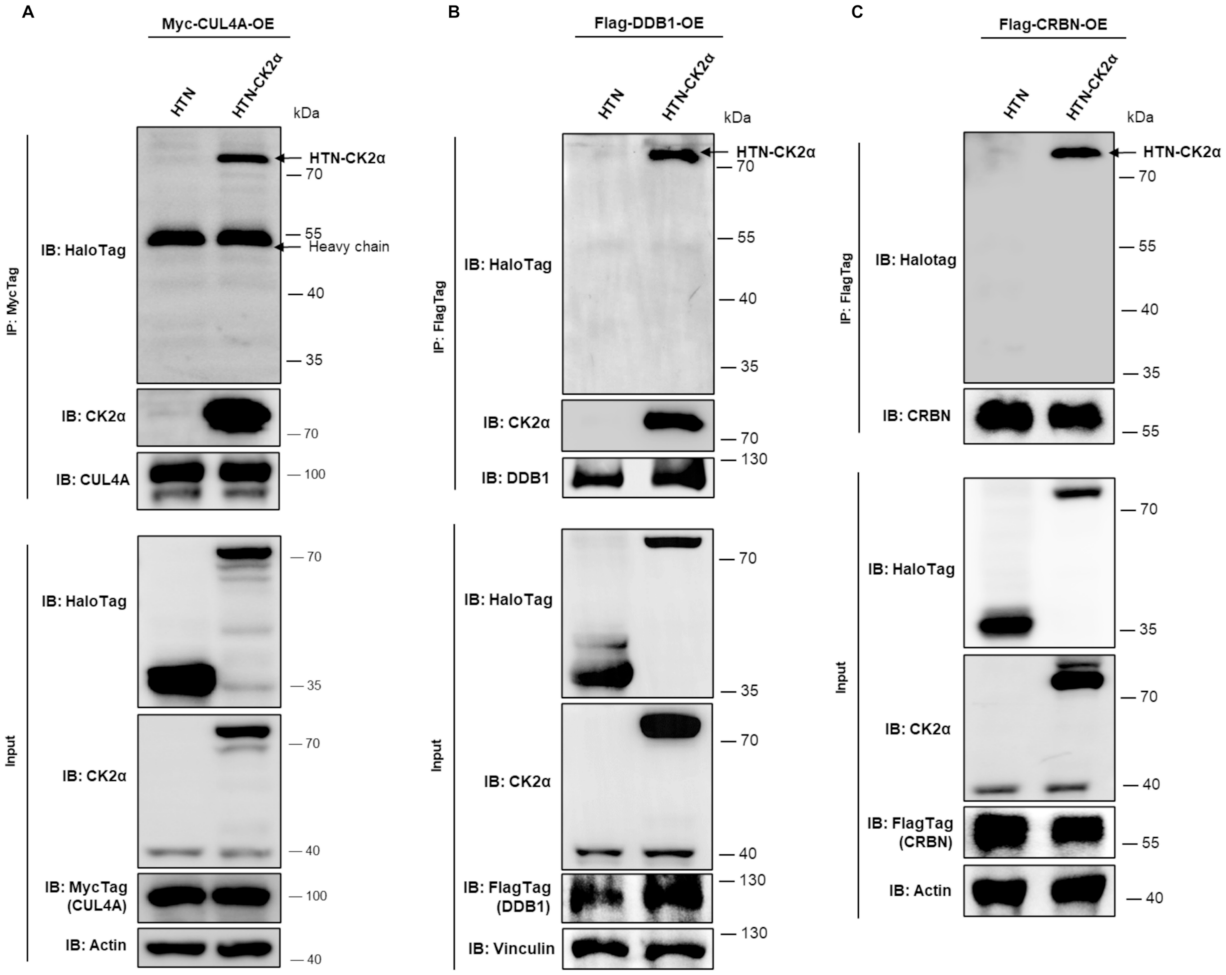
CRL4^CRBN^ E3 components interact with CK2α. (A–C) Co-IP showed that CK2α interacts with CUL4A, DDB1, and CRBN when coexpressed in HeLa cells. HTN-CK2α or control HTN (no CK2α) were coexpressed with each Flag- or Myc-tagged E3 component as indicated. IP was *via* the tag, and the presence of CK2α in precipitates was detected by anti-HaloTag antibody (for HTN-CK2α).

To further validate the relationship between the CRL4^CRBN^ complex and CK2α degradation, we conducted knockdown experiments of these CRL4^CRBN^ components and assessed the effects on the endogenous CK2α stability. We found that knockdown of any CRL4^CRBN^ component significantly increased endogenous CK2α by approximately 1.5-fold (Figure 6A,B and Supporting Figure 4). Furthermore, knocking down any CRL4^CRBN^ component significantly rescued CK2α degradation in a 36 h CHX chase assay (Figure 6C,D). These findings strongly suggest that the CRL4^CRBN^ E3 ubiquitin ligase complex can recognize CK2α as a substrate, working as a key mediator of CK2α ubiquitin-proteasome degradation.

**Figure 6:**
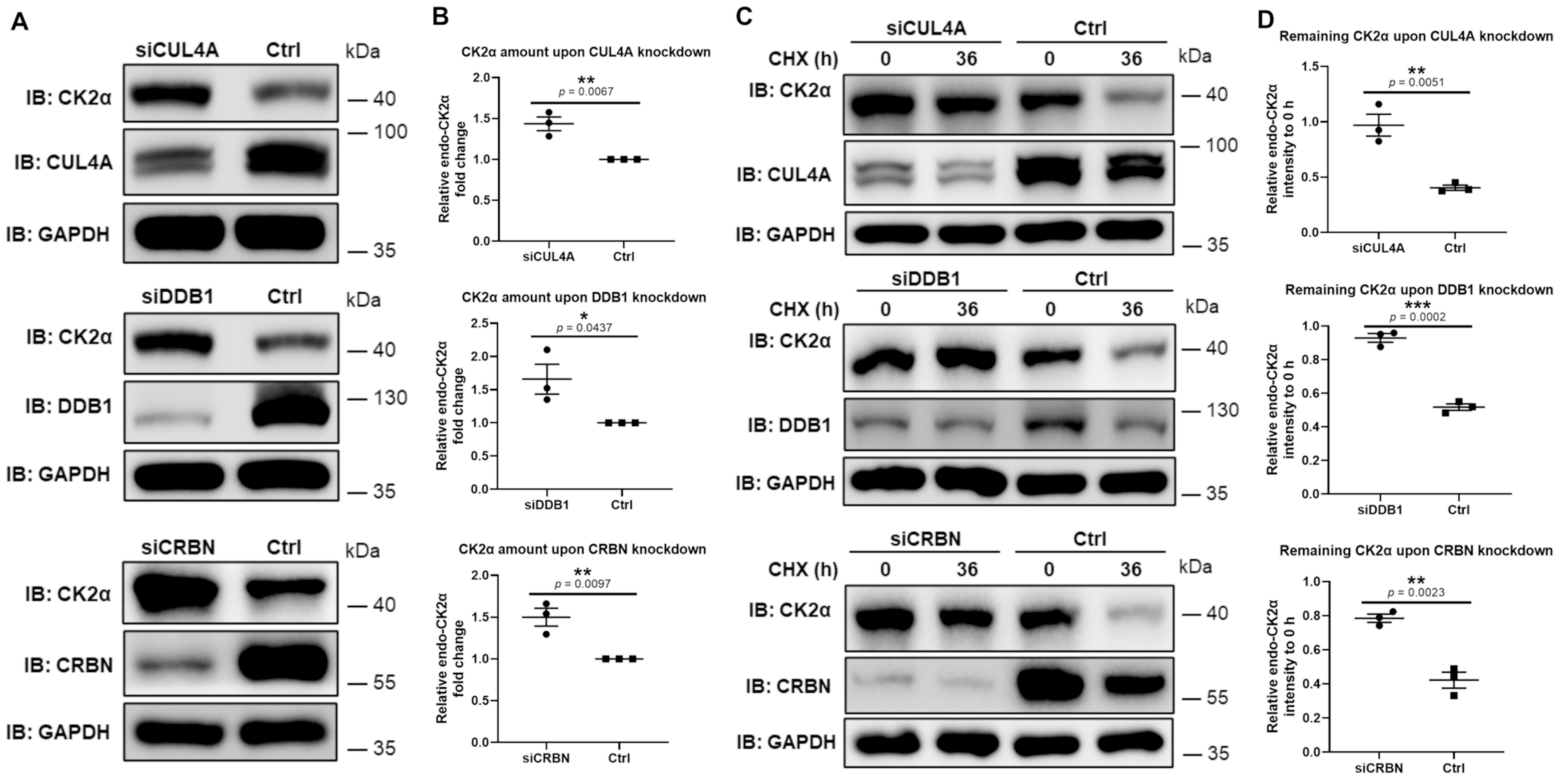
Knockdown of CUL4A/DDB1/CRBN rescues CK2α degradation. (A) Knockdown of CUL4A/DDB1/CRBN increased the CK2α level. HeLa cells were transfected with siRNA for 36 h accordingly, and the protein levels of CUL4A/DDB1/CRBN, CK2α, and GAPDH were assessed by Western blot. (B) Quantification of the relative endogenous CK2α level (CK2α signal normalized to GAPDH) fold change, normalized to the scrambled siRNA control (Ctrl). (C) Knockdown of CUL4A/DDB1/CRBN rescued CK2α degradation. Transfection was performed as above, followed by 36 h of CHX treatment before lysis and analyzing endogenous CK2α level. (D) Quantification of the remaining relative endogenous CK2α (CK2α signal normalized to GAPDH) after 36 h of CHX treatment, normalized to the 0 h relative CK2α level. Error bars represent the mean (SD) from *n* = 3 biologically independent experiments. Statistical significance for the quantifications was determined using an unpaired Student’s *t*-test. *p < 0.05; **p < 0.01; ***p < 0.001.

### Targeted O-GlcNAcylation of CK2α Increases Interaction with CRBN

Based on these results, we hypothesized that the O-GlcNAcylation plays an important role in how the CRL4^CRBN^ complex recognizes CK2α. To check this, we overexpressed HTN-CK2α (WT or S347A), Flag-CRBN, and fOGT in living cells. After 8 h of the OGTAC-1 treatment, the O-GlcNAcylation level of HTN-CK2α^WT^ was induced. Co-IP showed that the amount of CRBN bound to CK2α significantly increased by around 2-fold compared to that of the DMSO treatment (Figure 7A). HTN-CK2α^S347A^, however, kept lacking the O-GlcNAcylation and less interaction with CRBN, regardless of the OGTAC-1 treatment (Figure 7A). Furthermore, with the presence of OGTAC-1, co-IPed fOGT (HA-Tag) was detected, where only the CK2α with higher O-GlcNAcylation level showed more interaction with CRBN. This excluded the possibility that the induced proximity between CK2α and OGT led to more CK2α-CRBN interaction. Besides, *in vitro* pull-down assay also showed that CRBN exhibited a stronger interaction with HTN-CK2α^WT^ than HTN-CK2α^S347A^ (Supporting Figure 5).

**Figure 7:**
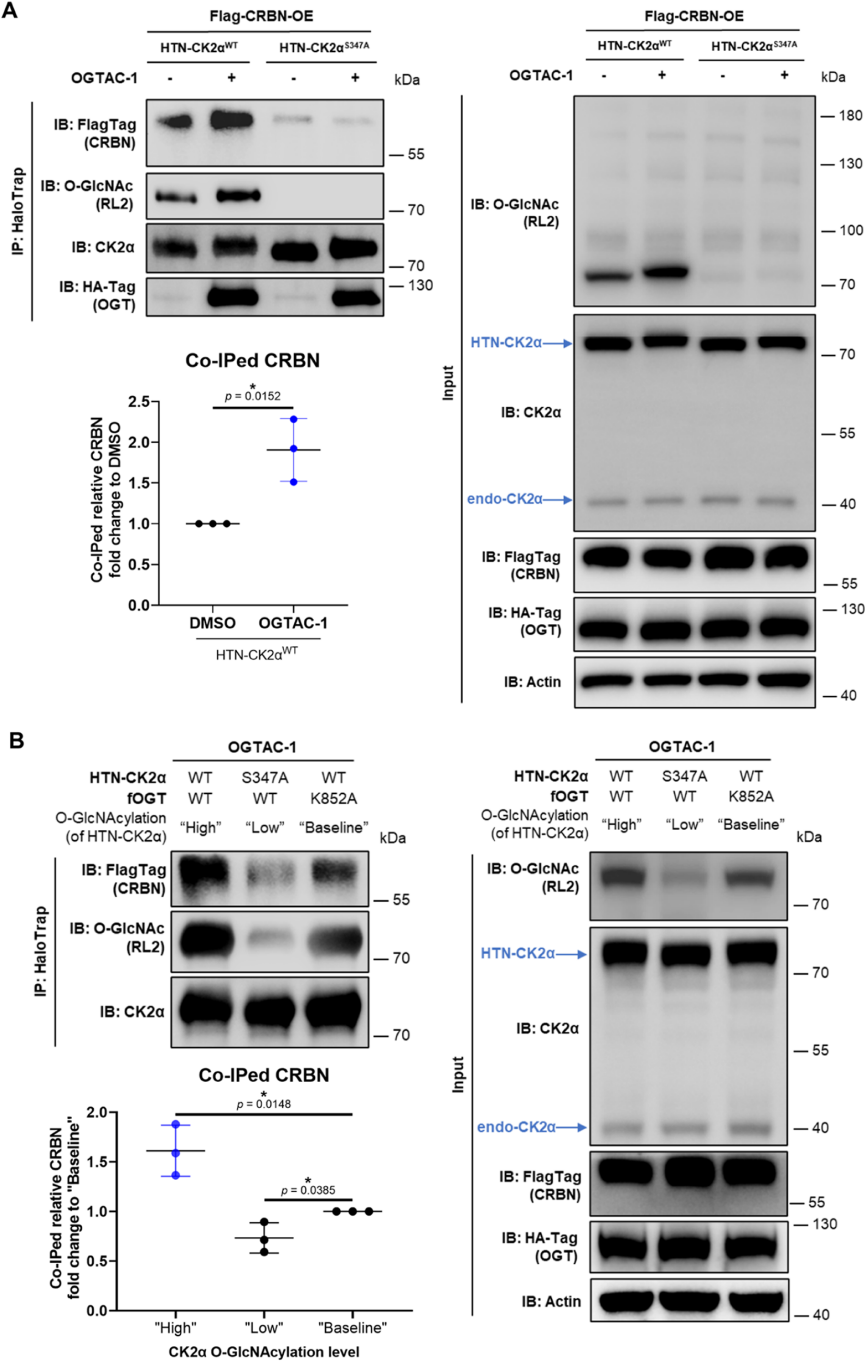
Targeted O-GlcNAcylation increases the CK2α-CRBN interaction. (A) Targeted O-GlcNAcylation of CK2α by OGTAC-1 (100 nM) for 8 h significantly increased CK2α-CRBN interaction. Flag-CRBN, HTN-CK2α (WT or S347A), and fOGT were overexpressed in cells. HaloTrap was used for IP, and anti-FlagTag was used for co-IP detection. For quantification, the relative level of the co-IPed CRBN (FlagTag signal normalized to CK2α IP) fold changes of HTN-CK2α^WT^ were calculated and normalized to the DMSO control. (B) CK2α at a higher O-GlcNAcylation level exhibited stronger interaction with CRBN. Flag-CRBN, HTN-CK2α (WT or S347A), and fOGT (WT or K852A) were overexpressed in cells. The cells were then treated with 100 nM OGTAC-1 for 8 h. IP and co-IP were performed as described above. For quantification, relative levels of the co-IPed CRBN (FlagTag signal normalized to CK2α IP) were calculated, normalized to that of the “Baseline” group (HTN-CK2α^WT^ and fOGT^K852A^ expressed) as the fold changes. Error bars represent the mean (SD) from *n* = 3 biologically independent experiments. Statistical significance for the quantifications was determined using an unpaired Student’s *t*-test. *p < 0.05.

To further confirm the role of CK2α O-GlcNAcylation in the CK2α-CRBN interaction, CRBN co-IP levels were compared across three HTN-CK2α groups with various levels of O-GlcNAcylation: (1) “High” (HTN-CK2α^WT^ + active fOGT^WT^), (2) “Low” (O-GlcNAcylation-deficient HTN-CK2α^S347A^ + active fOGT^WT^), and (3) “Baseline” (HTN-CK2α^WT^ + inactive fOGT^K852A^), all treated with OGTAC-1 (Figure 7B). As shown before, HTN-CK2α^WT^ in the “Baseline” group represented the baseline O-GlcNAcylation level (Supporting Figure 2B). Significantly, with the rate of O-GlcNAcylation of CK2α increasing, more co-IPed CRBN was detected. These findings shed light on how O-GlcNAcylation crosstalks with ubiquitination on CK2α—the modification enhances the interaction between CK2α and the CRL4^CRBN^ E3 ligase, thereby promoting CK2α’s ubiquitination and proteasomal degradation. However, although CK2α O-GlcNAcylation enhanced CRBN co-IP, it remains unclear whether this modification directly mediates their interaction. This question is the focus of ongoing investigations in our laboratory.

Nevertheless, these results reveal a mechanism in which adding an O-GlcNAc moiety on CK2α at Ser347 marks the protein for proteasomal degradation *via* the CRL4^CRBN^ ubiquitin ligase. This represents a crosstalk between O-GlcNAcylation and the ubiquitin-proteasome system.

### Targeted O-GlcNAcylation of CK2α Alters Downstream Phosphorylation

To understand the consequences of modulating the CK2α activity *via* O-GlcNAcylation, we examined downstream signaling outputs. Although CK2α O-GlcNAcylation does not alter its catalytic activity as a kinase, it can alter CK2α’s substrate selectivity.^8,19^ Through phospho-proteomics, a series of proteins were identified, whose phosphorylation was affected by changes in CK2α O-GlcNAcylation.^8^ Notably, some of these proteins, such as PFKP, have not been recognized as direct substrates of CK2α. PFKP, a crucial protein in glycolysis, has been reported to be associated with a poor prognosis in glioblastoma. PFKP Ser386 phosphorylation, which is catalyzed by Akt, plays an important role in this process.^30^ Akt is an established substrate of CK2α and a key protein in the PI3K-Akt signaling pathway. CK2α can phosphorylate Akt at Ser129, Thr308, and Ser473.^11,13,30^

Using OGTAC-1, we investigated the above and other site-specific phosphorylation events and found that CK2α O-GlcNAcylation alters downstream phosphorylation. After treatment with OGTAC-1 (10/100/1000 nM, effective concentrations for O-GlcNAcylation induction), phosphorylation of different CK2α substrates was detected. Significantly, an increase of Akt S129 and Akt S473 phosphorylation (pS129 and pS473) by about 2-fold, and an increase of PFKP S386 phosphorylation (pS386) by about 1.5-fold, were observed upon 8 h OGTAC-1 treatment (Figure 8A–D). This OGTAC-1-induced upregulation of the phosphorylation effect was lacking in the O-GlcNAcylation-deficient HTN-CK2α^S347A^ (Figure 8A and Supporting Figure 6). These indicated that the alternations of downstream phosphorylation were caused by targeted O-GlcNAcylation of CK2α, but not the induced proximity.

**Figure 8:**
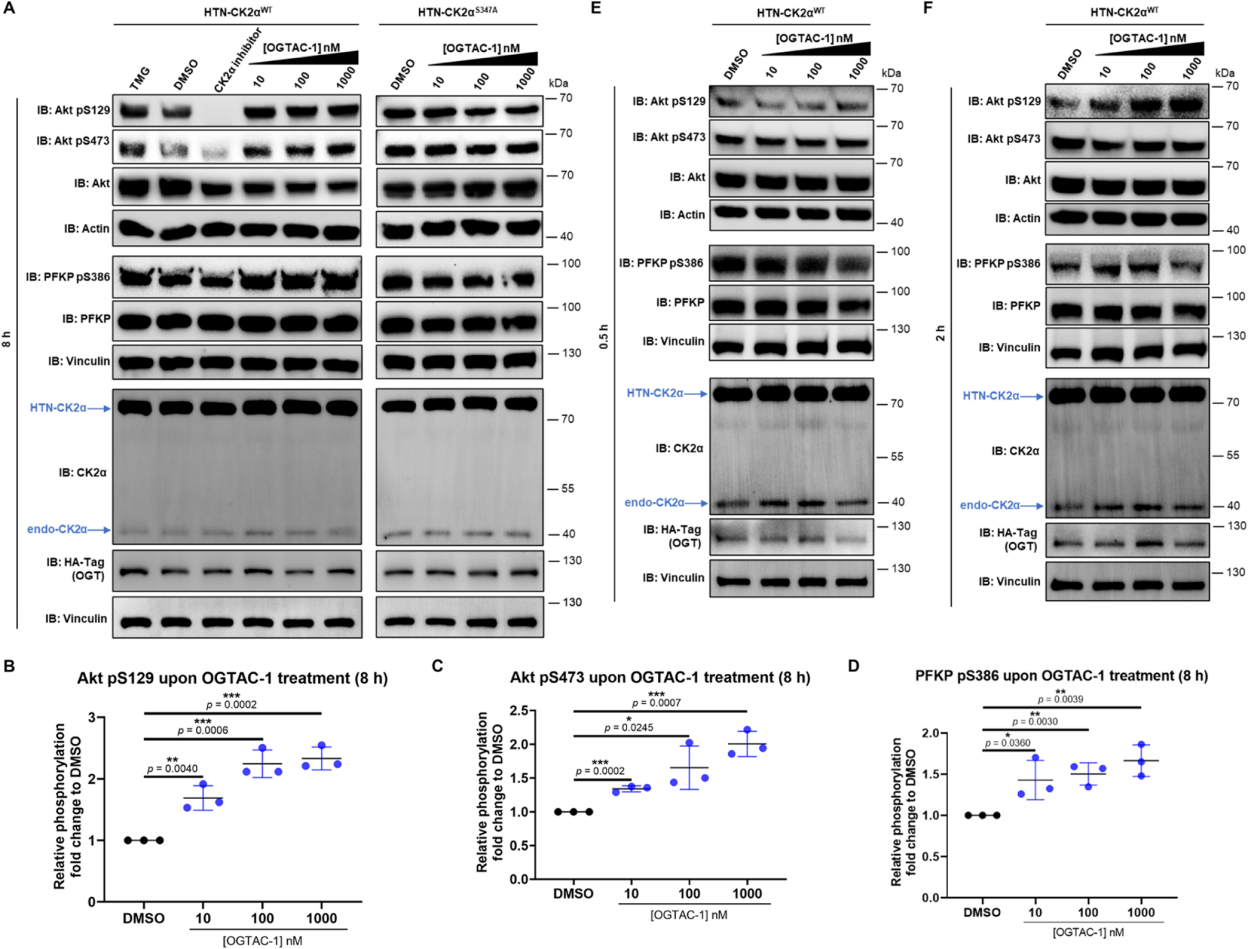
Targeted O-GlcNAcylation of CK2α alters downstream phosphorylation. (A) Targeted O-GlcNAcylation of CK2α by OGTAC-1 at 8 h significantly increased the phosphorylation levels of Akt Ser129, Akt Ser473, and PFKP Ser386. This effect was not observed in the O-GlcNAcylation-deficient S347A CK2α mutant (quantification shown in Supporting Figure 6). TMG (the O-GlcNAcylation positive control) and CK2α inhibitor (SGC–CK2–1) were treated in parallel as controls. (B–D) Quantification of relative phosphorylation (site-specific phosphorylation normalized to original protein level) fold changes from (A), normalized to DMSO treatment. Error bars represent the mean (SD) from *n* = 3 biologically independent experiments. Statistical significance for each quantification was determined using an unpaired Student’s *t*-test. *p < 0.05; **p < 0.01; ***p < 0.001; ns, not significant. (E, F) OGTAC-1 treatment increased Akt pS129 by 2 h, whereas Akt pS473 did not change at these early times (0.5 or 2 h). Besides, OGTAC-1 treatment did not lead to early change in PFKP pS386 at 0.5 or 2 h.

Additionally, 8 h CK2α inhibitor SGC–CK2–1 at high (1000 nM) and low (10 nM) concentration cotreatment with OGTAC-1 showed a large or minor decrease on Akt pS129, Akt pS473, and PFKP pS386, while the latter could be reversed by OGTAC-1 (Supporting Figure 7). This further validated the altered downstream phosphorylation of Akt and PFKP was dependent on OGTAC-1-induced CK2α O-GlcNAcylation.

To investigate OGTAC-1 kinetics, time-dependent (0.5 and 2 h) phosphorylation of these three sites was detected. Akt pS129 started to show activation by OGTAC-1 from 2 h, while Akt pS473 and PFKP pS386 did not (Figure 8E,F). This is in line with previous report that PFKP pS386 is closely related to Akt pS473.^30^

As for other CK2α-related downstream events, OGTAC-1 treatment did not change the signal of pan-CK2α substrate phosphorylation compared to DMSO (Supporting Figure 8A), which is consistent with previous reports that O-GlcNAcylation does not affect CK2α’s catalytic activity.^19^ Regarding the checked CK2α substrates, site-specific phosphorylation of Akt T308, NFκB P65 S529, STAT3 S727, and PTEN S370 did not show changes after treating with OGTAC-1 (Supporting Figure 8B–D). Notably, TMG largely reduced PTEN S370 phosphorylation (Supporting Figure 8D). This probably stemmed from TMG’s disruption of global O-GlcNAcylation, which may interfere with crosstalk between the O-GlcNAcylation and other PTMs. These confirm that CK2α downstream phosphorylation is not globally upended, while only specific sites are affected, reinforcing a targeted effect rather than general kinase hyperactivation.

We also linked the CK2α ubiquitination to its downstream phosphorylation. CRBN knockdown (48 h) and overexpression (48 h) led to a significant increase and decrease, respectively, of Akt pS129 and pS473 (Supporting Figure 9A–C). Adding the CK2α inhibitor erased phosphorylation on these two sites, indicating that the observed phosphorylation changes could probably occur because of the CK2α protein amount change related to CRL4^CRBN^.

While the O-GlcNAcylation of CK2α triggers its proteasomal degradation (as we illustrated by the CHX assay before), OGTAC-1 treatment within 8 h did not reduce the total amount of HTN-CK2α in cell (Figure 8A). Meanwhile, O-GlcNAcylation might alter CK2α’s substrate preference or signaling function in the short term, before degradation takes full effect, leading to the counterintuitive enhancement in the CK2α downstream phosphorylation of specific sites on Akt and PFKP.

## Conclusions

Using OGTAC to induce CK2α O-GlcNAcylation at Ser347, we demonstrate that targeted O-GlcNAcylation of CK2α by OGTAC-1 triggers its ubiquitin-proteasome degradation as a substrate of the CRL4^CRBN^ E3 complex and activates downstream phosphorylation. Specifically, our study suggests that targeted O-GlcNAcylation at Ser347 selectively modulates CK2α substrate engagement and local signaling preferences without broadly enhancing catalytic activity. This transient shift in substrate specificity briefly boosts downstream phosphorylation, such as Akt (pS129 and pS473) and PFKP (pS386), before observable degradation of O-GlcNAcylated CK2α. Our study aligns with previous findings related to CK2α stability,^19^ E3 ligases of CK2α,^27^ and the altered downstream phosphoproteome by CK2α O-GlcNAcylation.^8^

Notably, our results highlight important differences between targeted O-GlcNAcylation and traditional global perturbation methods. Previously, researchers primarily employed OGT and OGA inhibitors for O-GlcNAcylation studies.^31–33^ However, this approach disrupted global O-GlcNAcylation and potentially resulted in data ambiguities. For example, using the OGT inhibitor ST045849, Yu et al. reported that CK2α O-GlcNAcylation enhances protein stability.^34^ In contrast, both Tarrant et al. and Schwein et al. demonstrated that CK2α O-GlcNAcylation decreases its stability.^8,19^ Our findings unambiguously indicated that CK2α-specific O-GlcNAcylation decreases its stability and accelerates its proteasomal degradation.

Bulk O-GlcNAcylation modulators affect the global PTM level, leading to challenges in interpreting the downstream effects. In contrast, targeted strategies like OGTACs offer unique advantages as they allow for the dissection of the direct effects of protein-specific O-GlcNAcylation without the confounding factors introduced by bulk O-GlcNAcylation perturbation.

By addressing this technical gap, our study provides direct evidence of the crosstalk between CK2α O-GlcNAcylation and the ubiquitin-proteasome pathway and how its downstream phosphorylation is altered. This not only sets a precedent that the O-GlcNAcylation can serve as a degradation signal for proteins but also answers how this PTM alters CK2α function. These findings raise the possibility that modulating O-GlcNAcylation on specific oncoproteins like CK2α could be a strategy to control their stability and signaling, providing a potential new avenue for therapeutic intervention.

Looking forward, OGTACs can be applied to probe the role of O-GlcNAcylation on other proteins, potentially unveiling new O-GlcNAc-driven regulatory mechanisms and validating novel targets for therapeutic degradation *via* glycosylation-regulated pathways.

## Methods

### Cell Culture, DNA Constructs, and Reagents

Experiments were performed using the cancerous cervical tumor cell line HeLa, or HEK293T (kind gifts from Prof. Alfred Sze-Lok Cheng, CUHK), or the colon cancer cell line HCT116 (kind gifts from Prof. Kenneth Kin-Wah To, CUHK). Cells were cultured and maintained at 37 °C with 5% CO_2_ in Dulbecco′s modified Eagle′s medium (DMEM, Gibco-Invitrogen) supplemented with 10% fetal bovine serum (Gibco-Invitrogen) and 1% penicillin and streptomycin.

For plasmids, FKBP12^F36V^-HA-OGT (fOGT), pHTN vector, and HTN-CK2α, HTN-CK2α^S347A^ were obtained as previously described.^6^ fOGT^K852A^ was mutated from fOGT using Mut Express MultiS Fast Mutagenesis Kit V2 (Vazyme, C215). Primers were synthesized by Tsingke Gene. Flag-CRBN, Flag-DDB1, Myc-CUL4A, and FlagTag vector plasmids were purchased from Addgene (Addgene plasmid # 107380, 19918, 19951, and 210342).

Transient transfection was conducted according to the manufacturer’s protocol using Lipo2000 transfection reagent (AboRo, RL0401). OGTAC-1 was synthesized as previously described.^6^ OGTAC-1, Thiamet G (TMG, Bidepharm, BD571819), cycloheximide (CHX, Sigma-Aldrich, 239763-M), MG132 (MedChemExpress, HY-13259), chloroquine (CQ, MedChemExpress, HY-17589A), and CK2α inhibitor SGC–CK2–1 (MedChemExpress, HY-139004) were dissolved in DMSO (Sigma-Aldrich, D2650) to desired concentration and applied to cells.

### Plasmid Ratio Optimization for Transfection

1 × 10^5^ HeLa cells (1 × 10^5^) were seeded into 12-well plates. 0.5 μg of HTN-CK2α, with or without 0.5 μg/0.25 μg/0.1 μg/0.05 μg/0.025 μg/0.01 μg/0.005 μg (representing the transfection ratio of HTN-CK2α:fOGT = 1:1, 1:0.5, 1:0.2, 1:0.1, 1:0.05, 1:0.02, 1:0.01, and HTN-CK2α only) were transfected for 24 h. The samples were then processed and analyzed by Western blot as described in the Western Blot and Antibodies section part below.

### Chemoenzymatic Labeling and Mass Shift Assay

3 × 10^6^ HeLa cells were seeded in a 10 cm dish for each condition and then transfected with HTN-CK2α WT or S347A and fOGT. Cells were then treated with TMG (10 μM), DMSO, or OGTAC-1 (100 nM) for 8 h before lysis. The assay was then carried out as previously described and the samples were analyzed by Western blot.^6^

### Immunoprecipitation (IP) and co-IP

3 × 10^6^ HeLa or 2 × 10^6^ HEK293T cells were seeded in a 10 cm dish for each IP reaction. Transfection was performed at 50–60% confluency. After 24 h, media were replaced by fresh warm complete DMEM containing DMSO or probes for the desired duration. Then cells were washed by PBS and completely lysed using 600 μL of RIPA buffer (for IP, ThermoFisher, 89900) or IP lysis buffer (for co-IP, ThermoFisher, 87787), both containing 1× protease inhibitor cocktail (MedChemExpress, HY-K0010), 1× phosphatase inhibitor cocktail (MedChemExpress, HY-K0021), UniversalBenzo Nuclease (Vazyme, RM1022–01), and 20 μM TMG. The supernatant lysate was extracted by centrifuging at 15,000 rpm for 15 min at 4 °C and then applied to pre-equilibrated protein A/G magnetic beads (MedChemExpress, HY-K0202) which had already bound desired antibody, anti-Flag magnetic beads (MedChemExpress, HY-K0207), anti-MycTag magnetic beads (MedChemExpress, HY-K0206), or HaloTrap Magnetic Agarose (Proteintech, #otma) according to manufacturer’s protocol. After gentle rotation at 4 °C overnight, the beads were washed according to the manufacturer’s protocol and boiled in 80 μL of 2× SDS-loading buffer (Bio-Rad, #161–0737) at 95 °C for 5 min for elution. To detect IPed and co-IPed proteins, the eluted samples were directly applied to Western blot analysis.

### *In Vitro* Pull-Down Assay

5 × 10^6^ HEK293T cells were seeded in T225 flasks. Flag-CRBN and HTN-CK2α (WT or S347A) plasmids were separately transfected to different flasks. After 48 h, the cells were lysed and separated from the lysate by anti-Flag magnetic beads and Halo-Link resin followed by washing, respectively. The Flag-CRBN was then eluted using 0.15 M glycine (Meryer, M19902, pH = 2.5) as the manufacturer’s protocol and incubated with the on-resin HTN-CK2α (WT or S347A) at 4 °C overnight. After washing 5 times, the beads were boiled in 80 μL 2× SDS-loading buffer at 95 °C for 5 min for elution. To detect IPed HTN-CK2α (WT or S347A) and the pulled-down Flag-CRBN, the eluted samples were directly applied to Western blot analysis.

### Western Blot and Antibodies

For general whole cell lysate samples, 5 × 10^5^ HeLa cells seeded and treated in 6-well plates were lysed using 150 μL of complete RIPA buffer, collected, and spun down as described above. Bicinchoninic acid (BCA; ThermoFisher, 23225) assay was performed to adjust the cell lysate to a final concentration of 2 mg mL^–1^, and then the samples were denatured by adding final 1× SDS-loading buffer, and heating at 95 °C for 5 min. Equal amounts of protein samples for each batch were loaded for sodium dodecyl sulfate-polyacrylamide gel electrophoresis (SDS-PAGE, gels made using One-Step PAGE Gel Fast Preparation Kit (8%/12%, Vazyme, E302–01/E304–01)) and blotted with corresponding antibodies, with molecular weight indicated by 180 kDa prestained protein markers (Vazyme, MP102–01). Analysis and quantifications of Western blot results were performed using Image Lab Software Version 6.1 (BioRad) or ImageJ for windows.

Primary antibodies: Anti-RL2 (Abcam, ab2739; 1:1000 for WB), anti-CK2α (Cell Signaling Technology, 2656; 1:1000 for WB), anti-CK2β (ABclonal, A14722; 1:1000 for WB), anti-ubiquitin (Santa Cruz Biotechnology, sc-8017; 1:1000 for WB), anti-β-actin (ABclonal, AC026 or AC004; 1:100000 or 1:3000 for WB), anti-OGT (Cell Signaling Technology, 24083; 1:1000 for WB), anti-GAPDH (Santa Cruz Biotechnology, sc-47724; 1:3000 for WB), anti-Vinculin (Santa Cruz Biotechnology or ABclonal, sc-73614 or A2752; 1:1000 or 1:50000 for WB), anti-HaloTag (Promega, G9211; 1:1000 for WB), anti-CRBN (Cell Signaling Technology, 71810; 1:1000 for WB), anti-DDB1 (Fortis, A300–462A; 1:5000 for WB), anti-CUL4A (Fortis, A300–739A; 1:8000 for WB), anti-PFKP (Cell Signaling Technology, 8164; 1:1000 for WB), anti-PFKP phospho-Ser386 (Signalway, SAB616; 1:1000 for WB), anti-MycTag (MedChemExpress, HY-P80232; 1:3000 for WB), anti-FlagTag (MedChemExpress, HY-P80111; 1:8000 for WB), anti-HA-Tag (Vazyme, RA1004; 1:1500 for WB), anti-Akt1 (MedChemExpress, HY-P80007; 1:1000 for WB), anti-Akt1 phospho-S129 (Abcam, ab133458; 1:1000 for WB), anti-Akt phospho-Thr308 (UpingBio, YP-Ab-14409; 1:1000 for WB), anti-Akt1 phospho-Ser473 (MedChemExpress, HY-P80788; 1:1000 for WB), anti-NFκB P65 (Cell Signaling Technology, 8242; 1:1000 for WB), anti-NFκB P65 phospho-Ser529 (MedChemExpress, HY-P80470; 1:1000 for WB), anti-STAT3 (MedChemExpress, HY-P80902; 1:1000 for WB), anti-STAT3 phospho-Ser727 (MedChemExpress, HY-P80281; 1:1000 for WB), anti-PTEN (MedChemExpress, HY-P80286; 1:1000 for WB), anti-PTEN phospho-Ser370 (Abcam, ab195056; 1:1000 for WB), anti-Phospho-CK2 Substrate [(pS/pT)­DXE] MultiMab (Cell Signaling Technology, 8738; 1:1000 for WB).

Secondary antibodies: Anti-rabbit HRP-linked antibody (Cell Signaling Technology, 7074; 1:8000 for WB), anti-mouse HRP-linked antibody (Cell Signaling Technology, 7076; 1:8000 for WB; besides, we recommend a lower ratio of dilution, 1:20000, for better detection of the RL2 signal of highly O-GlcNAcylated proteins from the whole cell lysate), anti-rabbit DyLight 488 antibody (ThermoFisher, 35522; 1:5000 for WB), anti-mouse DyLight 488 antibody (ThermoFisher, 35503; 1:5000 for WB).

### Cycloheximide (CHX) Chase Assay

5 × 10^5^ HeLa cells were seeded into 6-well plates. Transfection of HTN-CK2α or HTN-CK2α^S347A^, and fOGT, was performed as described above. After 24 h, media were replaced freshly, and the cells were treated with 1 μM OGTAC-1 or DMSO, both for 8 h. Then, fresh complete media containing 50 μg/mL CHX were applied to the cells for 0, 3, 6, 12, 24, and 36 h before being lysed, normalized, and denatured as mentioned above. The cell samples were then used to analyze the remaining relative HTN-CK2α level, fOGT level (by anti-HA-Tag), and β-actin/vinculin level *via* Western blot.

1 × 10^5^ HCT116 cells were seeded into 12-well plates. 0.5 μg HTN-CK2α (WT or S347A) plasmids were first transfected for 24 h. Then, the medium was replaced and 0.5 μg fOGT plasmids was transfected for another 24 h. After the transfection, pretreatment of OGTAC-1 (or DMSO) was performed as described above. Then, fresh complete media containing 50 μg/mL CHX were applied to the cells for 12 h before being lysed. Sample preparation was performed as described above. The cell samples were then used to analyze the remaining relative HTN-CK2α level, fOGT level (by anti-HA-Tag), and vinculin level *via* Western blot.

### Degradation Rescue Assay

3 × 10^5^ HeLa cells were seeded into 12-well plates. Transfection of HTN-CK2α and fOGT was performed as described above. After 24 h, the cells were treated with fresh media containing either 25 μM MG132 or 100 μM CQ, or DMSO for 2 h. Then fresh media containing 1 μM OGTAC-1 or DMSO were added for 8 h. Next, fresh complete media containing 50 μg/mL CHX were applied for 0 or 12 h. The cells were then lysed with 100 μL of complete RIPA buffer, normalized, and made into denatured samples as described above. Remaining relative HTN-CK2α level, the OGT level, and the β-actin level were compared between w/o MG132 or CQ pretreated groups *via* Western blot.

### CK2α Ubiquitination Assay

3 × 10^6^ HeLa cells were seeded in a 10 cm dish for each group in the pull-down assay. HTN-CK2α (WT or S347A) and fOGT plasmids were cotransfected for 24 h. The cells were first pretreated by 25 μM MG132 for 2 h. Then, fresh media containing either 100 nM of OGTAC-1 or DMSO for both HTN-CK2α^WT^ or HTN-CK2α^S347A^ were replaced for 8 h. After the treatment, cells from each plate were then lysed and collected using 600 μL specially prepared RIPA lysis buffer, containing 20 μM TMG, 1× protease inhibitor cocktail, 1× phosphatase inhibitor cocktail, nuclease, 5 mM Ethylenediaminetetraacetic acid (EDTA, Meryer, M20959), 100 mM N-Ethylmaleimide (NEM, MedChemExpress, HY-D0843; BidePharm, BD144276). Then, HTN-CK2α was trapped and eluted using HaloTrap Magnetic Agarose (Proteintech, #otma) according to manufacturer’s protocol. Afterward, HTN-CK2α’s ubiquitination level was analyzed through Western blot and anti-ubiquitin antibody as described. The HaloTrap elution sample blots were heated in boiled ddH_2_O for 10 min before blocking. This procedure was optimized from published protocols.^35^

To check CK2α ubiquitination change upon the CRL4^CRBN^ E3 component overexpression, 1.5 × 10^6^ HeLa cells were seeded in each 6 cm dish. Any of the E3 components (Flag-DDB1, Myc-CUL4A and Flag-CRBN) or the FlagTag vector and HTN-CK2α were transfected altogether for 48 h. Then, the cells were treated with 25 μM MG132 for 4 h before collecting. The following steps were performed according to the descriptions above.

### siRNA Knockdown

Control siRNAs and human DDB1/CUL4A/CRBN siRNAs were synthesized by GenePharma. The siRNA sequences for human siDDB1 (sense): (1) 5′-AACGGCUGCGUGACCGGACAC-3′;^36^ (2) 5′-CAGCCACACAGAGAUGGAACAUGAA-3′. The siRNA sequences for human siCUL4A (sense): (1) 5′-GAAGAUUAACACGUGCUGGTT-3′;^36^ (2) 5′-GAAUCUCUGAUAGACAGAGACUAUA-3′. The siRNA sequences for human siCRBN (sense): (1) 5′-CCCAGACACUGAAGAUGAAAU-3′;^37^ (2) 5′-AAGACCAGGAUAGUAAAGAAGCCAA-3′. The sequence for the scrambled negative control siRNA (sense): 5′-UUCUCCGAACGUGUCACGUTT-3′. 2 × 10^5^ HeLa cells were seeded in 12-well plates, and were then transfected with 20 pmol siRNA using siRNA-Mate transfection reagent (GenePharma, G04002) or Lipo2000 (Vazyme, TL201) for 36 h according to manufacturer’s protocol. Then the cells were collected or further treated for the CHX chase assay as described above, before being lysed and analyzed by Western blot.

### Phosphorylation Assay

To analyze site-specific phosphorylation level change of downstream proteins (Akt Ser129/Ser473/Thr308, PFKP Ser386, NF-κB P65 Ser529, PTEN Ser370, and STAT3 Ser727), cotransfected HeLa cells seeded in 12-well plates were treated with 10/100/1000 nM OGTAC-1 for 8 h. Samples treated by OGTAC-1 at 0.5 and 2 h were also collected for Akt pS129, Akt pS473, and PFKP pS386 time-dependent analysis. SGC–CK2–1 (1 μM), TMG (10 μM), and DMSO were treated as controls. In the meantime, the same concentration series of OGTAC-1 or DMSO were applied to HTN-CK2α^S347A^ transfected cells as controls. Cell samples were prepared as described above in the Western blot part, with a final protein concentration of 3 mg/mL. Protein phosphorylation level and protein amount were determined using Western blot and the antibodies described above accordingly. For Akt-related blots, detections were made either in the order of Akt pSer129, Akt pSer473, Akt, and β-actin, or Akt pThr308, Akt, and β-actin, followed by 10 min stripping using Antibody Stripping Buffer (Vazyme, E701). The same methods were applied for other proteins in the order of site-specific phosphorylation antibody—original protein antibody—housekeeper protein antibody followed by stripping. The same samples were also used for global CK2-substrate phosphorylation determination followed by vinculin detection. For the CK2α inhibition reverse assay in Supporting Figure 7, 10 nM or 1000 nM SGC–CK2–1 was cotreated with OGTAC-1 at above concentrations for 8 h before cell lysis.

### Protein Structure Modeling

To predict HTN-CK2α structure, its full amino acid sequence was input into Boltz-1, running with the default parameters.^38^ Domains were highlighted by using PyMOL Open Source.

### Protein Purification and *In Vitro* Kinase Activity ADP-Glo Assay

To obtain HTN-CK2α, HTN-CK2α^S347A^, and HTN proteins, first, 6 × 10^6^ HeLa cells were seeded in 15 cm dishes. Then, 10 μg of any of the three plasmids was transfected in each dish for 48 h before collecting. Next, the HaloTag proteins were purified using Halo-Link Resin (Promega, G1912) according to the manufacturer’s protocol. To verify the purified proteins, 2× loading buffer was applied to part of the resin products, eluting 95 °C for 5 min. The supernatant was analyzed by SDS-PAGE and Western blot. Finally, HTN-CK2α’s kinase activity was detected directly on-resin, using Kinase-Lumi Max Luminescent Kinase Assay Kit (Beyotime, S0158S) according to manufacturer’s protocol, with or without the Casein Kinase 2 Substrate Peptide (MedChemExpress, HY-P3815). Concentration of this substrate was optimized from previous studies.^22^

## Supplementary Material



## Notes

Additional data figures, experimental details and materials (PDF)

## References

[1] Saha A., Bello D., Fernández-Tejada A. (2021). Advances in
chemical
probing of protein O-GlcNAc glycosylation: structural role and molecular
mechanisms. Chem. Soc. Rev..

[2] Pratt M. R., Vocadlo D. J. (2023). Understanding and
exploiting the roles of O-GlcNAc
in neurodegenerative diseases. J. Biol. Chem..

[3] Cheng S. S., Mody A. C., Woo C. M. (2024). Opportunities for
Therapeutic Modulation
of O-GlcNAc. Chem. Rev..

[4] Fardini Y., Dehennaut V., Lefebvre T., Issad T. (2013). O-GlcNAcylation: a
new cancer hallmark?. Front. Endocrinol..

[5] Wu C., Li J., Lu L., Li M., Yuan Y., Li J. (2024). OGT and OGA:
Sweet guardians of the genome. J. Biol. Chem..

[6] Ma B., Khan K. S., Xu T., Amada J. X., Guo Z., Huang Y., Yan Y., Lam H., Cheng A. S.-L., Ng B. W.-L. (2024). Targeted Protein O-GlcNAcylation Using Bifunctional
Small Molecules. J. Am. Chem. Soc..

[7] Zhu Y., Hart G. W. (2023). Dual-specificity
RNA aptamers enable manipulation of
target-specific O-GlcNAcylation and unveil functions of O-GlcNAc on
β-catenin. Cell.

[8] Schwein P. A., Ge Y., Yang B., D’Souza A., Mody A., Shen D., Woo C. M. (2022). Writing
and Erasing O-GlcNAc on Casein Kinase 2 Alpha
Alters the Phosphoproteome. ACS Chem. Biol..

[9] Ramirez D. H., Aonbangkhen C., Wu H.-Y., Naftaly J. A., Tang S., O’Meara T. R., Woo C. M. (2020). Engineering a Proximity-Directed
O-GlcNAc Transferase for Selective Protein O-GlcNAcylation in Cells. ACS Chem. Biol..

[10] Roffey S. E., Litchfield D. W. (2021). CK2 Regulation:
Perspectives in 2021. Biomedicines.

[11] Halloran D., Pandit V., Nohe A. (2022). The Role of Protein Kinase CK2 in
Development and Disease Progression: A Critical Review. J. Dev. Biol..

[12] Meggio F., Pinna L. A. (2003). One-thousand-and-one
substrates of protein kinase CK2?. FASEB J..

[13] Di
Maira G., Salvi M., Arrigoni G., Marin O., Sarno S., Brustolon F., Pinna L. A., Ruzzene M. (2005). Protein kinase
CK2 phosphorylates and upregulates Akt/PKB. Cell Death Differ..

[14] Guerra B. (2006). Protein kinase
CK2 subunits are positive regulators of AKT kinase. Int. J. Oncol..

[15] Liu J., Cao X. C., Xiao Q., Quan M. F. (2015). Apigenin inhibits
HeLa sphere-forming cells through inactivation of casein kinase 2α. Mol. Med. Rep..

[16] Zou J., Luo H., Zeng Q., Dong Z., Wu D., Liu L. (2011). Protein kinase
CK2α is overexpressed in colorectal cancer and modulates cell
proliferation and invasion via regulating EMT-related genes. J. Transl. Med..

[17] Kreppel L. K., Hart G. W. (1999). Regulation of a cytosolic and nuclear O-GlcNAc transferase.
Role of the tetratricopeptide repeats. J. Biol.
Chem..

[18] Bosc D. G., Slominski E., Sichler C., Litchfield D. W. (1995). Phosphorylation
of Casein Kinase II by p34^cdc2^: IDENTIFICATION OF PHOSPHORYLATION
SITES USING PHOSPHORYLATION SITE MUTANTS IN VITRO. J. Biol. Chem..

[19] Tarrant M. K., Rho H. S., Xie Z., Jiang Y. L., Gross C., Culhane J. C., Yan G., Qian J., Ichikawa Y., Matsuoka T., Zachara N., Etzkorn F. A., Hart G. W., Jeong J. S., Blackshaw S., Zhu H., Cole P. A. (2012). Regulation
of CK2 by phosphorylation and O-GlcNAcylation revealed by semisynthesis. Nat. Chem. Biol..

[20] Ruan H.-B., Nie Y., Yang X. (2013). Regulation
of Protein Degradation by O-GlcNAcylation:
Crosstalk with Ubiquitination. Mol. Cell. Proteomics.

[21] Prudent, R. ; Sautel, C. F. ; Moucadel, V. ; Laudet, B. ; Filhol, O. ; Cochet, C. In vitro and in vivo assays of protein kinase CK2 activity. In Methods in Enzymology; Elsevier, 2010; Vol. 485, pp 597–610. 21050938 10.1016/B978-0-12-381296-4.00031-2

[22] Gratz A., Götz C., Jose J. (2010). A CE-based assay for human protein
kinase CK2 activity measurement and inhibitor screening. Electrophoresis.

[23] Ramirez D. H., Ge Y., Woo C. M. (2021). O-GlcNAc
Engineering on a Target Protein in Cells with
Nanobody-OGT and Nanobody-splitOGA. Curr. Protoc..

[24] Alteen M. G., Tan H. Y., Vocadlo D. J. (2021). Monitoring and modulating O-GlcNAcylation:
assays and inhibitors of O-GlcNAc processing enzymes. Curr. Opin. Struct. Biol..

[25] Sahu I., Glickman M. H. (2021). Proteasome in action:
substrate degradation by the
26S proteasome. Biochem. Soc. Trans..

[26] Kleiger G., Mayor T. (2014). Perilous journey: a tour of the ubiquitin-proteasome system. Trends Cell Biol..

[27] Wang H., Tang X., Liu Y. (2023). SlCK2α
as a novel substrate
for CRL4 E3 ligase regulates fruit size through maintenance of cell
division homeostasis in tomato. Planta.

[28] Chen H., Chen F., Liu N., Wang X., Gou S. (2018). Chemically
induced degradation of CK2 by proteolysis targeting chimeras based
on a ubiquitin–proteasome pathway. Bioorg.
Chem..

[29] Lee J., Zhou P. (2007). DCAFs, the
Missing Link of the CUL4-DDB1 Ubiquitin Ligase. Mol. Cell.

[30] Lee J.-H., Liu R., Li J., Zhang C., Wang Y., Cai Q., Qian X., Xia Y., Zheng Y., Piao Y., Chen Q., de Groot J. F., Jiang T., Lu Z. (2017). Stabilization
of phosphofructokinase 1 platelet isoform by AKT promotes tumorigenesis. Nat. Commun..

[31] Yang X., Qian K. (2017). Protein O-GlcNAcylation: emerging mechanisms and functions. Nat. Rev. Mol. Cell Biol..

[32] Lee B. E., Suh P.-G., Kim J.-I. (2021). O-GlcNAcylation
in health and neurodegenerative
diseases. Exp. Mol. Med..

[33] Parker M. P., Peterson K. R., Slawson C. (2021). O-GlcNAcylation
and O-GlcNAc Cycling
Regulate Gene Transcription: Emerging Roles in Cancer. Cancers.

[34] Yu Z., He H., Jiang B., Hu J. (2024). O-GlcNAcylation of CSNK2A1 by OGT
is Involved in the Progression of Colorectal Cancer. Mol. Biotechnol..

[35] Emmerich C. H., Cohen P. (2015). Optimising methods
for the preservation, capture and identification
of ubiquitin chains and ubiquitylated proteins by immunoblotting. Biochem. Biophys. Res. Commun..

[36] Bondar T., Kalinina A., Khair L., Kopanja D., Nag A., Bagchi S., Raychaudhuri P. (2006). Cul4A and
DDB1 Associate with Skp2
To Target p27Kip1 for Proteolysis Involving the COP9 Signalosome. Mol. Cell. Biol..

[37] Yang J., Huang M., Zhou L., He X., Jiang X., Zhang Y., Xu G. (2018). Cereblon suppresses
the lipopolysaccharide-induced
inflammatory response by promoting the ubiquitination and degradation
of c-Jun. J. Biol. Chem..

[38] Wohlwend, J. ; Corso, G. ; Passaro, S. ; Reveiz, M. ; Leidal, K. ; Swiderski, W. ; Portnoi, T. ; Chinn, I. ; Silterra, J. ; Jaakkola, T. ; Barzilay, R. Boltz-1 Democratizing Biomolecular Interaction Modeling bioRxiv 2024.

