# Supplementary material for: Targeted O‑GlcNAcylation of CK2α Triggers Its Ubiquitin-Proteasome Degradation and Alters Downstream Phosphorylation
Source: ACS Chem Biol. 2025 Jun 16;20(7):1646–59. doi: 10.1021/acschembio.5c00223 (PMC12281484; doi:10.1021/acschembio.5c00223)
Supplement: Supplementary file 1 [file cb5c00223_si_001.pdf]

Targeted O-GlcNAcylation of CK2α Triggers Its Ubiquitin-Proteasome Degradation and Alters Downstream Phosphorylation

Supporting Information

Tongyang Xu[1], Bowen Ma[1], Yuanpei Li[1], Zhihao Guo[1], Miaomiao Zhang[1], and Billy Wai-Lung Ng[1,2,3,4]*

1. Guangdong-Hong Kong-Macao Joint Laboratory for New Drug Screening, School of Pharmacy, The Chinese University of Hong Kong, Sha Tin, Hong Kong
2. Li Ka Shing Institute of Health Sciences, Faculty of Medicine, The Chinese University of Hong Kong, Sha Tin, Hong Kong
3. Gerald Choa Neuroscience Institute, The Chinese University of Hong Kong, Sha Tin, Hong Kong
4. Peter Hung Pain Research Institute, Faculty of Medicine, The Chinese University of Hong Kong, Sha Tin, Hong Kong

*: billyng@cuhk.edu.hk

Supplementary Figures

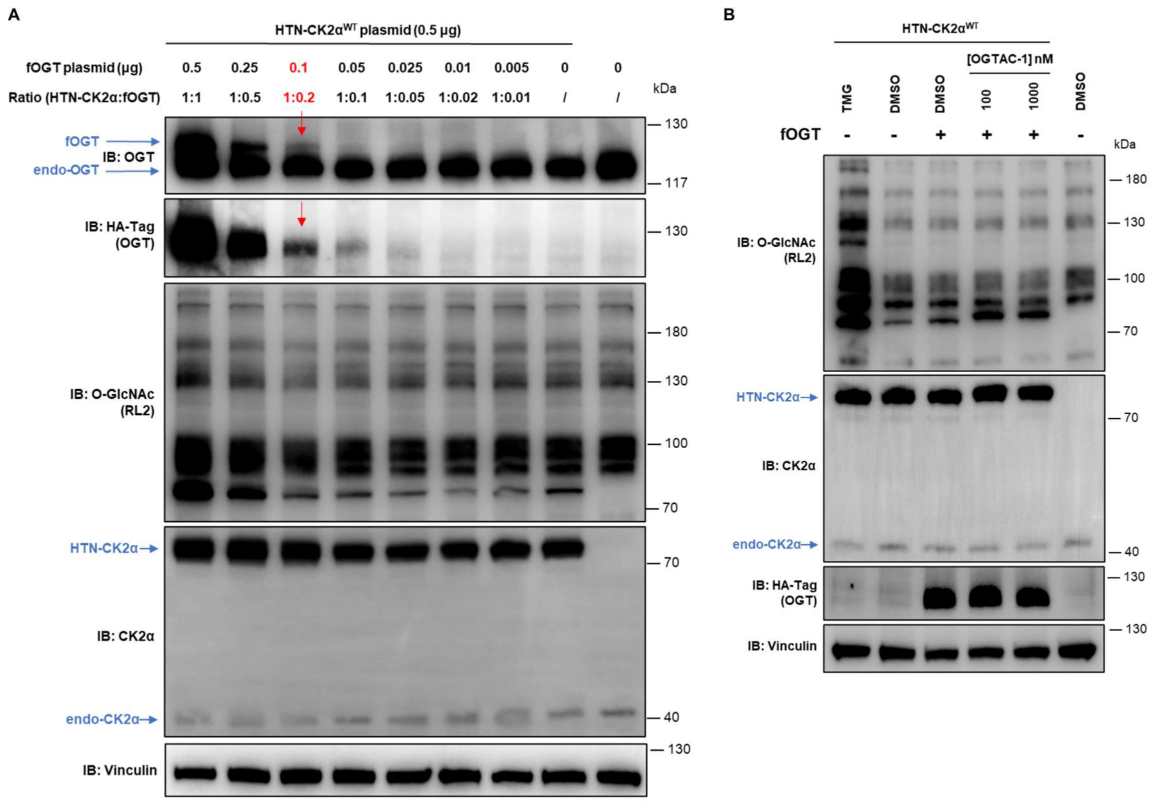

Supplementary Figure 1 Overexpression of FKBP-2×HA-OGT (fOGT) and N-terminal HaloTag CK2α (HTN-CK2α) in HeLa cells.

(A) Transfection ratio optimization of fOGT and HTN-CK2α. In a 12-well plate of HeLa cells, the plasmid amount of HTN-CK2α was set to be 0.5 μg for each well. A series of ratios of HTN-CK2α:fOGT were tried to determine an appropriate co-overexpression level: 1:1, 1:0.5, 1:0.2, 1:0.1, 1:0.05, 1:0.02, 1:0.01. The 1:0.2 ratio showed a moderate expression level of fOGT, and no obvious disturbance for O-GlcNAcylation of the whole proteome and HTN-CK2α. Using OGT antibody to detect fOGT co-overexpressed with HTN-CK2α, fOGT showed as a separate band at about 130 kDa from endogenous OGT, which could be detected by HA-Tag antibody. (B) With or without OGTAC-1, fOGT overexpression did not cause elevation of basic whole-proteome O-GlcNAcylation of HeLa cells. HeLa cells in 6-well plates were either transfected 1 μg HTN-CK2α plasmids, with or without 0.2 μg fOGT, or not. After 24 h transfection, thiamet-G (TMG) /OGTAC-1 (100 nM or 1000 nM) /DMSO were treated for 8 h, and then the cells were lysed for western blot analysis.

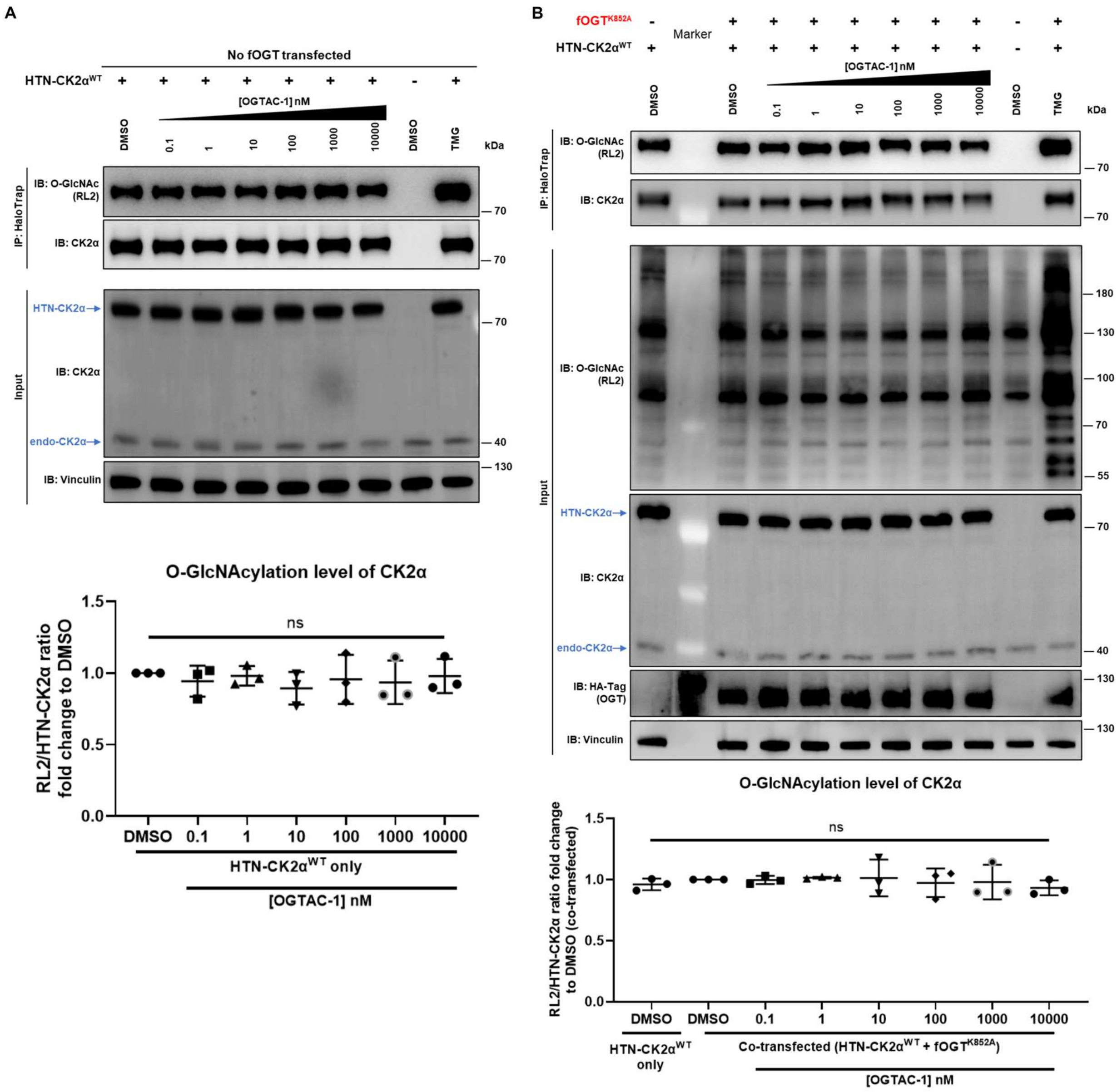

Supplementary Figure 2 OGTAC-1 did not induce O-GlcNAcylation of HTN-CK2α in the absence of fOGT or catalytically active fOGT.

(A) OGTAC-1 did not induce targeted O-GlcNAcylation of HTN-CK2α without fOGT. This was performed in the same condition as Figure 2B, but without fOGT overexpression. (B) OGTAC-1 did not induce targeted O-GlcNAcylation of HTN-CK2α with overexpression of the catalytically inactive fOGT^{K852A}. This was performed in the same condition as Figure 2B, but with overexpression of fOGT^{K852A} instead of fOGT^{WT}. Error bars represent the mean (SD) from *n* = 3 biologically independent experiments. Statistical significance was assessed using a one-way ANOVA. ns, not significant.

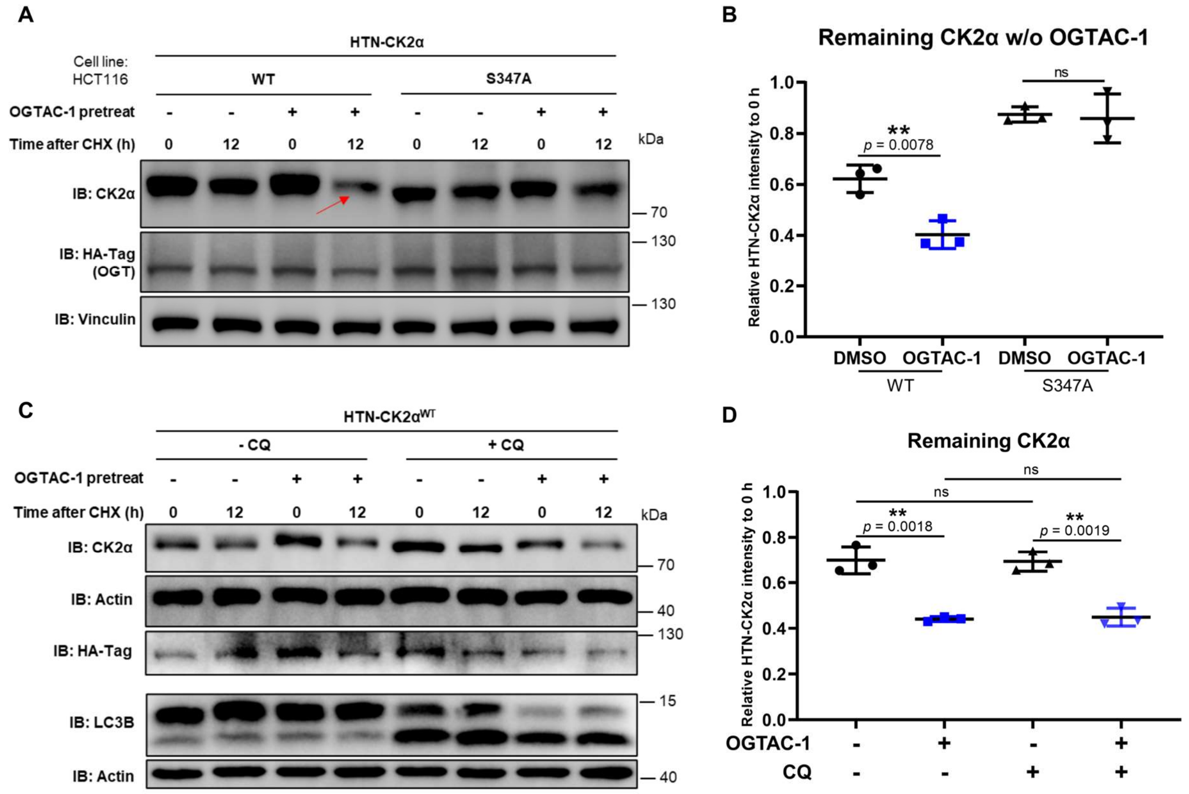

Supplementary Figure 3 The OGTAC-1-triggered degradation acceleration of CK2α was also observed in the HCT116 cell line, and the lysosomal degradation inhibitor chloroquine (CQ) did not rescue CK2α degradation.

(A) In HCT116 cells, after adding CHX for 12 h, only HTN-CK2α^{WT} with OGTAC-1 pre-treatment (1 μM) showed significantly more degradation. (B) Quantification of the remaining relative HTN-CK2α (CK2α normalized to Vinculin) after 12 h CHX treatment in (C), normalized to the 0 h level. (C) CQ did not increase CK2α level before and after a 12 h CHX chase, with or without OGTAC-1 (1 μM) treatment. (D) Quantification of the remaining relative HTN-CK2α (CK2α normalized to Actin) after 12 h CHX treatment in (C), normalized to the 0 h level. Error bars represent the mean (SD) from n = 3 biologically independent experiments. Statistical significance was assessed using an unpaired Student's t-test. **p < 0.01; ns, not significant.

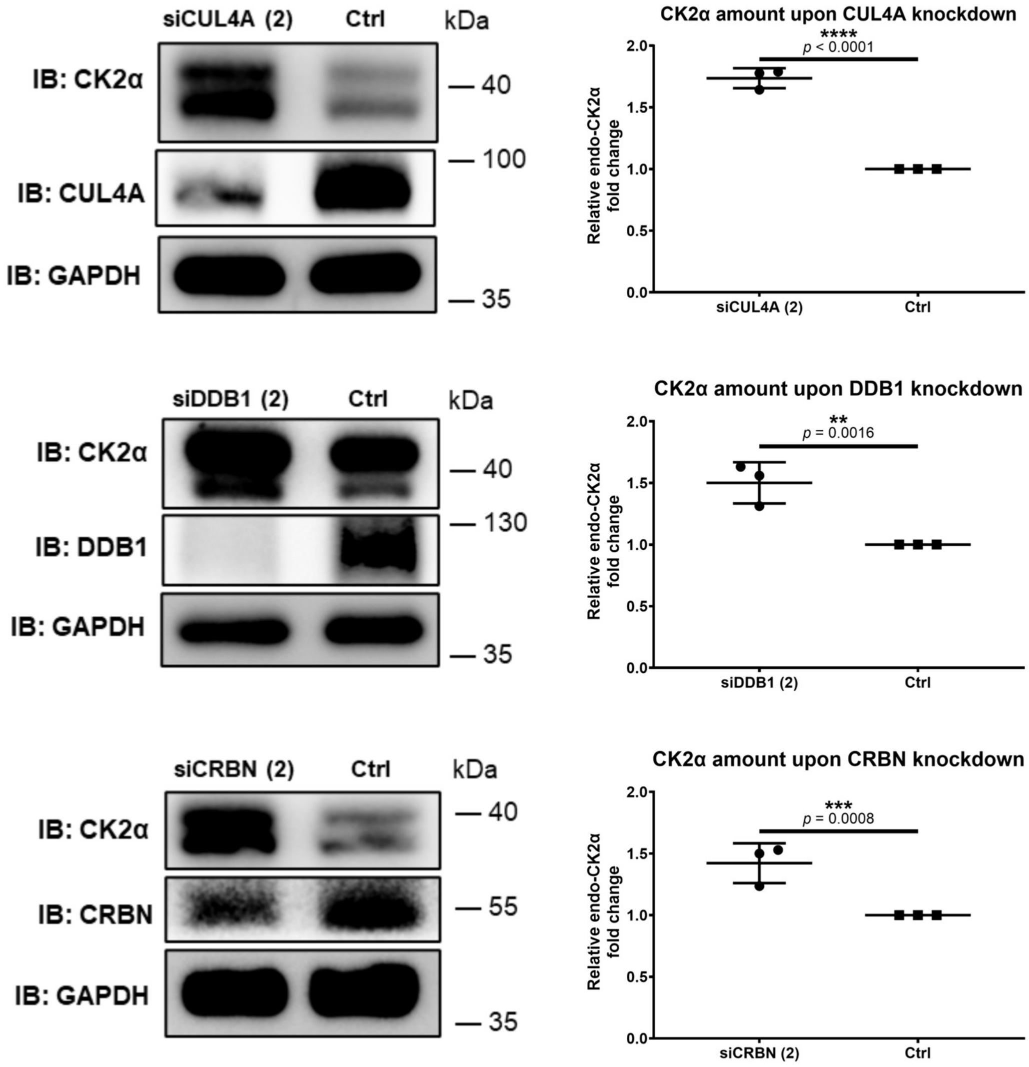

Supplementary Figure 4 Knockdown of CUL4A/DDB1/CRBN using a second siRNA sequence increased CK2α level.

(A) Knockdown of CUL4A/DDB1/CRBN increased CK2α level. Here the three siRNA for each protein were different from the one used in main text. HeLa cells were transfected with siRNA for 36 h accordingly, and the protein levels of CUL4A/DDB1/CRBN, CK2α, and GAPDH were assessed by western blot. (B) Quantification of relative endogenous CK2α level (CK2α normalized to GAPDH) fold change, normalized to siRNA control (Ctrl). Error bars represent the mean (SD) from $n = 3$ biologically independent experiments. Statistical significance for the quantifications was determined using an unpaired Student's *t*-test. $^{**}p < 0.01$; $^{***}p < 0.001$; $^{****}p < 0.0001$.

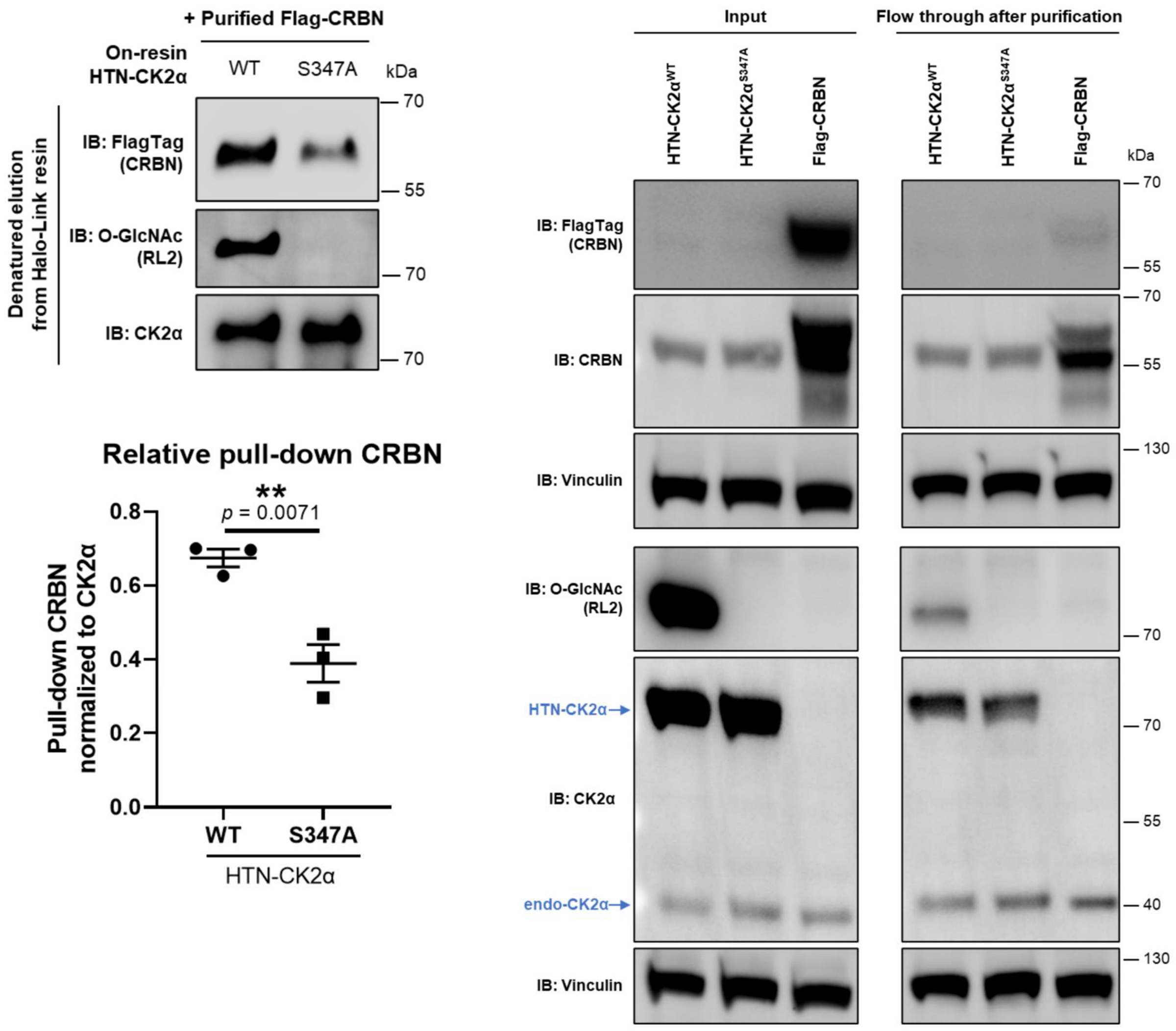

Supplementary Figure 5 CRBN interacted stronger with HTN-CK2α^{WT} than HTN-CK2α^{S347A} *in vitro*.

In the *in vitro* pull-down assay, the purified Flag-CRBN interacted stronger with HTN-CK2α^{WT} than HTN-CK2α^{S347A}, which cannot be O-GlcNAcylated. Flag-CRBN and HTN-CK2α (WT or S347A) were over-expressed in HEK293T cells for 48 h and purified. HTN-CK2α (WT or S347A) was IPed by HaloTrap, and the pulled-down Flag-CRBN was detected by anti-FlagTag. Relative level of the pulled-down Flag-CRBN (Flag-CRBN normalized to HTN-CK2α^{WT} or HTN-CK2α^{S347A}) was then quantified. Error bars represent the mean (SD) from n = 3 biologically independent experiments. Statistical significance for the quantifications was determined using an unpaired Student's t-test. **$p < 0.01$.

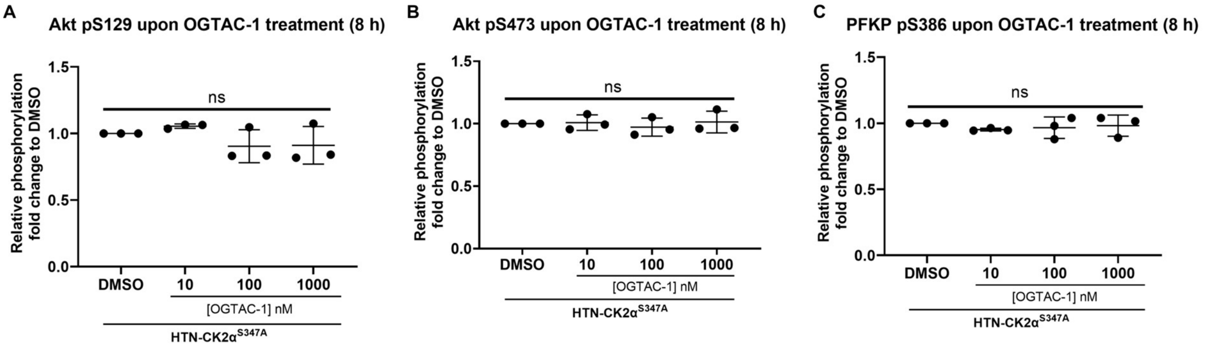

Supplementary Figure 6 Quantification of the blots in Figure 8A showing that OGTAC-1 did not induce Akt pS129, pS473, and PFKP pS386 when transfecting HTN-CK2α^{S347A} instead of HTN-CK2α^{WT}.

Quantification of relative phosphorylation (site-specific phosphorylation normalized to original protein level) fold changes from the blots in Figure 8A overexpressing HTN-CK2α^{S347A} instead of HTN-CK2α^{WT}, normalized to DMSO treatment. Error bars represent the mean (SD) from n = 3 biologically independent experiments. Statistical significance for each quantification was assessed using a one-way ANOVA. ns, not significant.

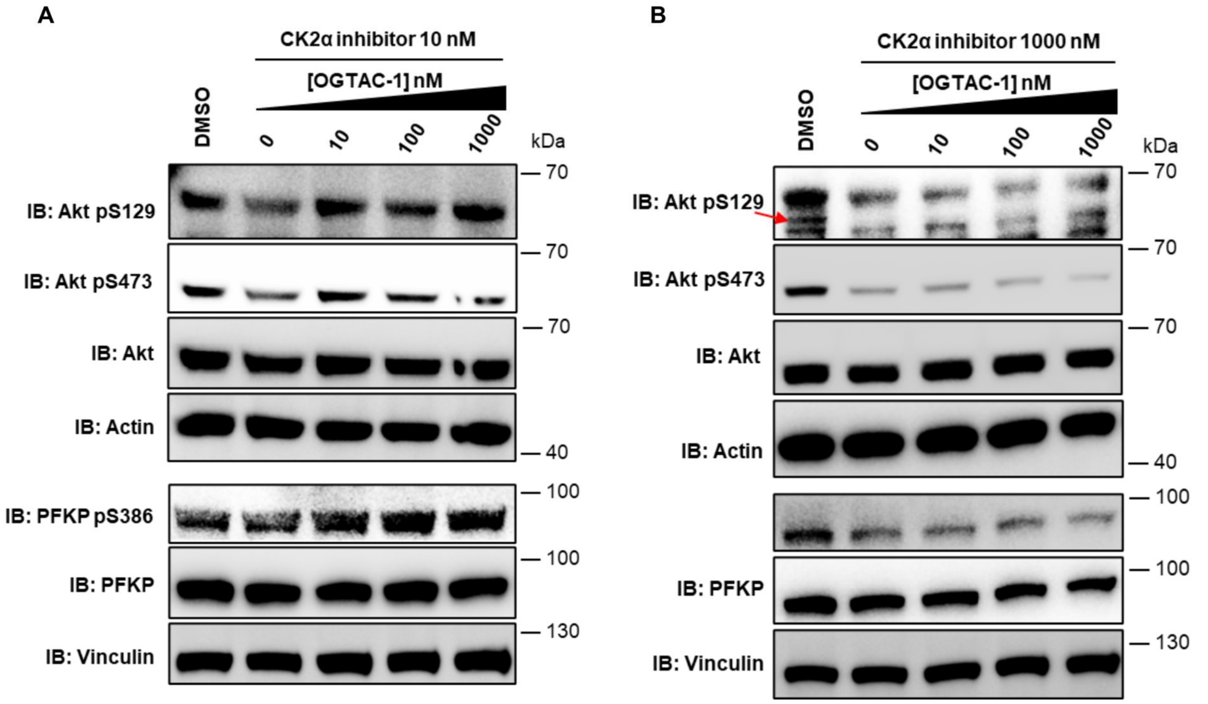

Supplementary Figure 7 Co-treatment of CK2α inhibitor (SGC-CK2-1) and OGTAC-1 further validated targeted O-GlcNAcylation of CK2α altered downstream phosphorylation.

(A) Treating low concentration (10 nM) CK2α inhibitor slightly inhibited Akt pS129, Akt pS473, and PFKP pS386, while this effect was reversed by co-treatment with OGTAC-1 (10/100/1000 nM). (B-D) Treating high concentration (1000 nM) CK2α inhibitor erased Akt pS129 and largely inhibited Akt pS473 and PFKP pS386, and this effect could not be reversed by OGTAC-1 co-treatment. All treatments were performed for 8 h.

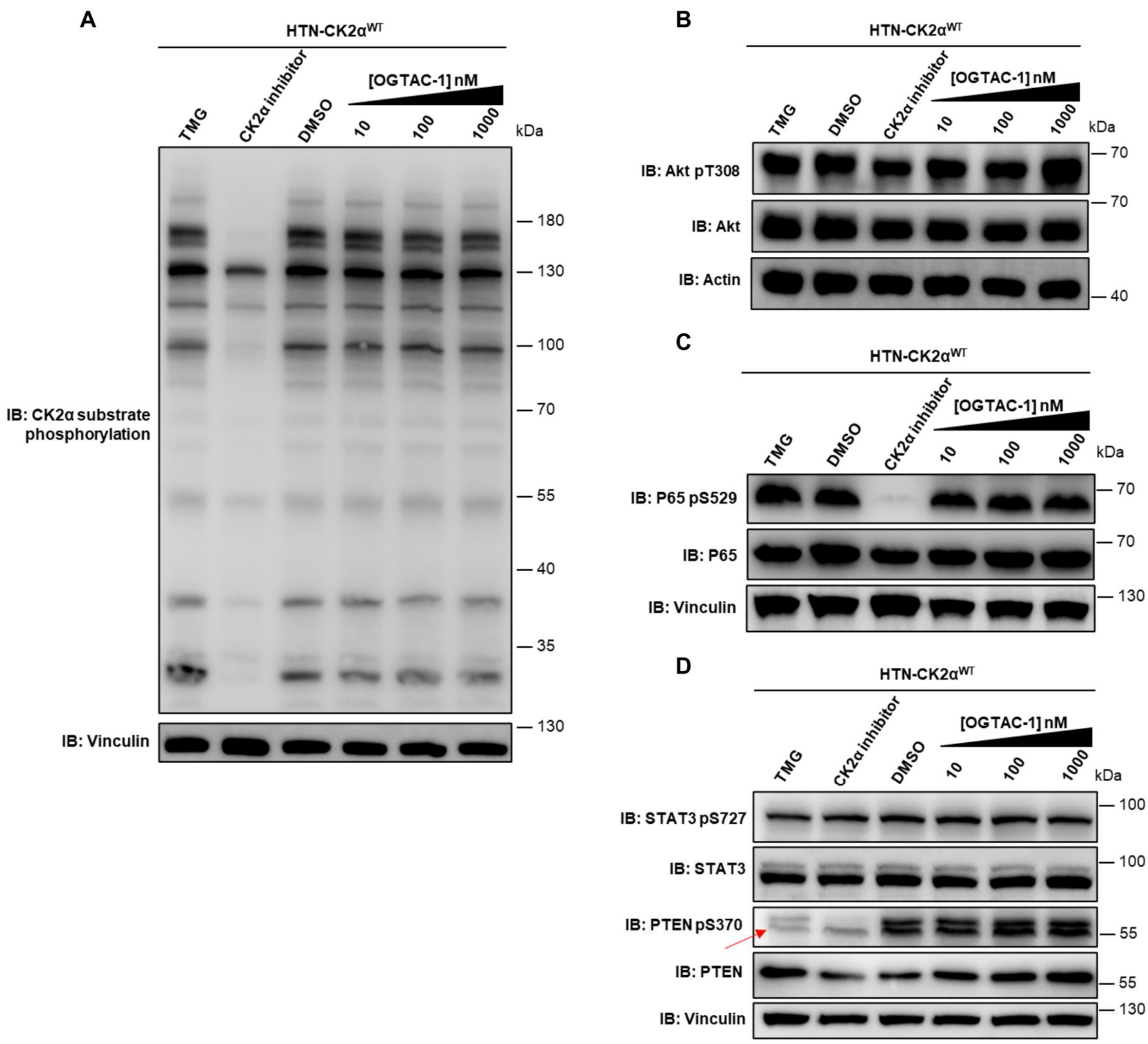

Supplementary Figure 8 Targeted O-GlcNAcylation of CK2α did not show changes in detecting pan-CK2α substrate phosphorylation, Akt pT308, NFκB P65 pS529, STAT3 pS727, or PTEN pS370.

(A) Targeted O-GlcNAcylation of CK2α by OGTAC-1 at 8 h did not show changes using CK2α pan-substrate phosphorylation antibody for detection. (B-D) Targeted O-GlcNAcylation of CK2α by OGTAC-1 at 8 h did not show changes in Akt pT308, NFκB P65 pS529, STAT3 pS727, or PTEN pS370. TMG (the O-GlcNAcylation positive control) and CK2α inhibitor (SGC-CK2-1) were treated in parallel as controls.

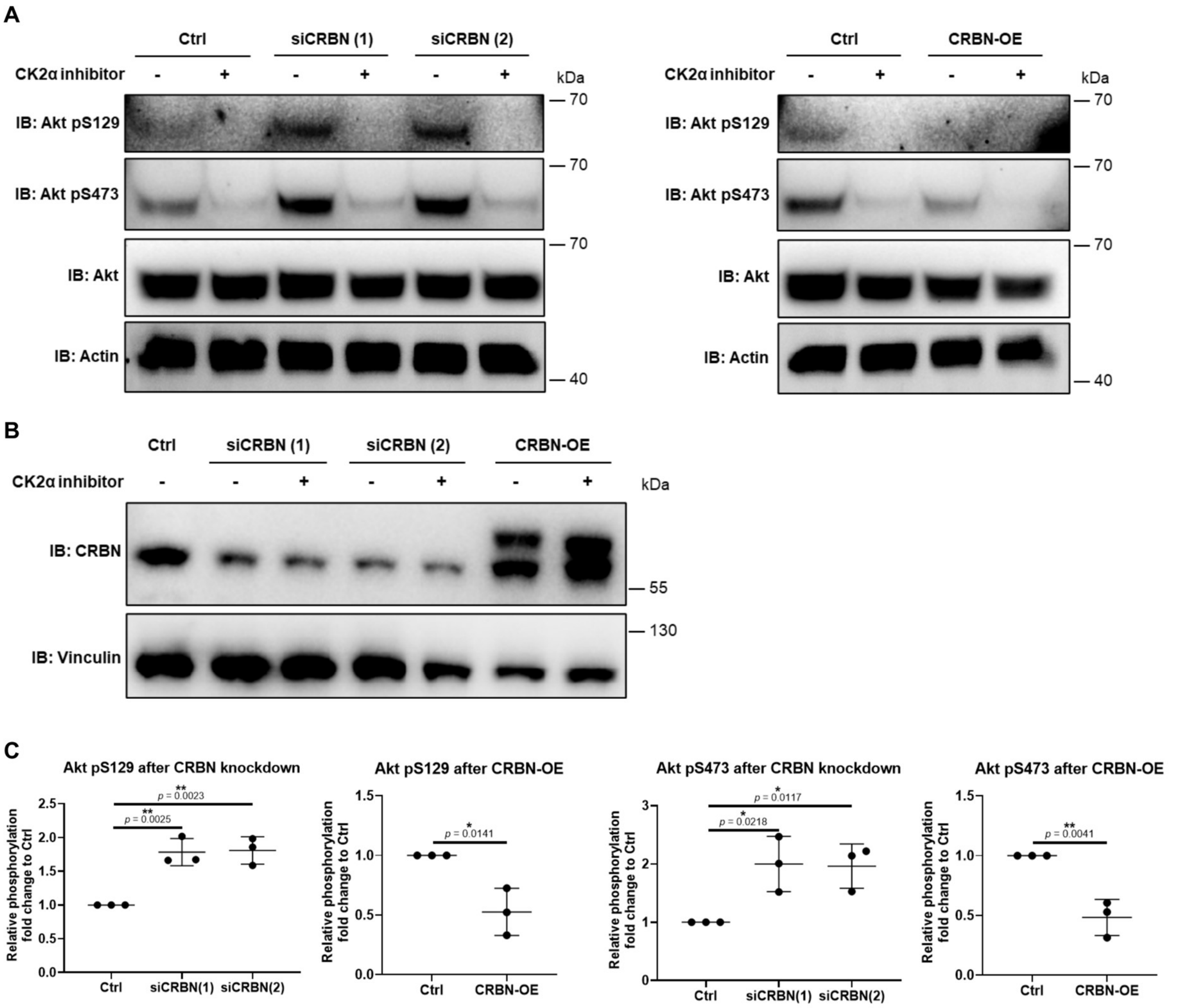

Supplementary Figure 9 Knockdown of CRBN increased Akt pS129 and pS473, and CRBN overexpression decreased them.

(A) Knockdown of CRBN increased Akt pS129 and pS473, and CRBN overexpression decreased them. For knockdown, HeLa cells were transfected with CRBN siRNAs for 36 h, where the scrambled siRNA was used as control. For overexpression, HeLa cells were transfected with Flag-CRBN for 36 h, where the FlagTag vector was used as control. For each condition, there was a CK2α inhibitor control (SGC-CK2-1 1 μM). Then the protein levels of CRBN, Akt pS129, Akt pS473, and Actin were assessed by western blot. (B) CRBN was either knocked down or overexpressed, compared to the original CRBN level. (C) Quantification of relative Akt pS129 and pS473 (phosphorylation signal normalized to Akt level) fold change, normalized to controls (Ctrl). Error bars represent the mean (SD) from n = 3 biologically independent experiments. Statistical significance for the quantifications was determined using an unpaired Student's *t*-test. *$p < 0.05$; **$p < 0.01$.
